# Clinical outcomes associated with adding pelvic correction manipulation to adjacent needling for lumbar disc herniation: a prospective comparative cohort study

**DOI:** 10.3389/fmed.2026.1936013

**Published:** 2026-08-24

**Authors:** Cheng Li, Jingyan Xu, Yinfei Xie, Xiaohua Liu, Xinjian Ma

**Affiliations:** 1 Department of Acupuncture and Moxibustion, Affiliated Hospital of Jiangnan University, Wuxi, Jiangsu, China; 2 Department of Cardiology, Rehabilitation and Convalescence Center of the Joint Logistic Support Force of the Chinese People’s Liberation Army, Tianjin, China; 3 Department of Traditional Chinese Medicine, Guangyi Subdistrict Community Health Service Centre, Wuxi, Jiangsu, China

**Keywords:** adjacent needling, cohort study, comparative effectiveness, lumbar disc herniation, pelvic correction manipulation, sagittal alignment

## Abstract

**Background:**

Lumbar disc herniation (LDH) commonly causes persistent pain, disability, and recurrence despite conservative care. Whether pelvic correction manipulation adds durable benefit to adjacent needling remains uncertain.

**Objective:**

This study aimed to evaluate the association of adding pelvic correction manipulation to adjacent needling with disability, pain, and recurrence in LDH.

**Methods:**

This single-center prospective comparative cohort study with retrospective long-term outcome ascertainment enrolled consecutive adults with symptomatic, imaging-confirmed LDH in Wuxi, China, from December 2019 to December 2020. Patients received adjacent needling plus pelvic correction manipulation or adjacent needling alone according to clinician–patient choice. The primary outcome was change in Oswestry Disability Index (ODI) at treatment completion. Secondary outcomes included pain, Japanese Orthopedic Association score, ODI response, recurrence, and adverse events through 24 months. Baseline-adjusted mixed-effects models, propensity-score overlap weighting, multiple imputation, and sensitivity analyses were used.

**Results:**

Among 208 patients, 106 received combined therapy and 102 adjacent needling alone. Combined therapy was associated with greater ODI reduction at treatment completion (adjusted difference, −3.40 points; 95% CI, -5.40 to -1.40; *P* = 0.001) and at 24 months (−4.40; 95% CI, −7.30 to −1.50; *P* = 0.003). At 24 months, ≥50% ODI improvement occurred in 58.9% versus 40.5% (adjusted RR, 1.41; 95% CI, 1.06–1.87), and recurrence in 23.6% versus 39.2% (adjusted HR, 0.55; 95% CI, 0.34–0.89). Exploratory alignment, mobility, and inflammatory findings also favored combined therapy. No serious treatment-related events occurred.

**Conclusion:**

Adding pelvic correction manipulation was associated with modestly better long-term disability outcomes and lower recurrence, but randomized trials are needed to confirm causality. Because treatment was not randomized, these findings should be read as associations rather than proof of a causal effect, and unmeasured confounding cannot be excluded.

## Introduction

Lumbar disc herniation (LDH) is a common degenerative spinal disorder in which nucleus pulposus material protrudes or extrudes through a weakened annulus fibrosus, producing mechanical compression and chemical irritation of adjacent neural structures. LDH presents with low back pain, radiating leg pain, sensory disturbance, restricted lumbar movement and, in severe cases, motor weakness. It predominantly affects working-age adults, with incidence highest during the third to fifth decades of life (1). Persistent symptoms may impair mobility, sleep, psychological well-being, work capacity and quality of life, while recurrent episodes can lead to repeated healthcare use and prolonged work absence (1). Although many patients improve with conservative management, incomplete functional recovery and recurrent radicular symptoms remain clinical concerns.

Management is guided by symptom severity, neurological status, imaging findings and response to conservative care. Urgent surgical evaluation is required in patients with progressive neurological impairment or cauda equina syndrome. In patients without an immediate surgical indication, treatment begins with nonoperative strategies, including activity modification, medication, exercise-based rehabilitation, physical therapy, acupuncture and manual therapy. Surgery can provide relief in selected patients, but operative treatment is neither necessary nor desirable for every patient (2). Conservative interventions that reduce pain, restore function, facilitate return to work and decrease recurrence remain central to LDH management (2). This approach is supported by current guidance. The 2023 World Health Organization guideline for non-surgical management of chronic primary low back pain, which provides broad contextual rather than herniation-specific direction, gives conditional recommendations in favor of needling therapies and spinal manipulative therapy, and a recent network meta-analysis of non-pharmacological interventions in Chinese adults reported that such therapies generally improve function and overall efficacy more than pharmacological treatment (3, 4). Disease-specific recommendations for lumbar disc herniation are considered in the Discussion.

Acupuncture is used as part of conservative care for LDH and disc-related sciatica. Its therapeutic effects may involve modulation of nociceptive processing, relaxation of paraspinal musculature and regulation of inflammatory activity. Adjacent needling is a technique used in Chinese practice in which a perpendicular needle is placed at the symptomatic site and paired with an obliquely directed needle whose tip converges toward it. This arrangement provides focused stimulation of painful lumbar, gluteal or lower-limb regions. Randomized evidence indicates that acupuncture can improve pain and function in patients with chronic sciatica caused by lumbar disc herniation (5). Nevertheless, needling may not adequately address coexisting lumbo-pelvic biomechanical dysfunction, and some patients continue to experience residual disability, restricted movement or recurrent symptoms (5).

These limitations have encouraged the integration of acupuncture with manual or manipulative interventions. Manual treatment may complement needling by reducing muscle guarding, improving joint and soft-tissue mobility and modifying abnormal loading across the lumbar spine and pelvis. A recent randomized trial involving 240 patients reported that acupuncture combined with manipulation produced greater short-term improvements in pain and lumbar function than acupuncture, manipulation or traction alone (6). However, available studies have generally used short follow-up periods, leaving the durability of improvement uncertain (6). Manual therapy protocols are also heterogeneous, and few studies have evaluated a defined pelvic correction approach with disability response, recurrence, additional treatment, return to work and surgery.

A further rationale for pelvic correction manipulation arises from lumbo-pelvic sagittal alignment. Pelvic incidence is a stable morphological parameter that influences sacral orientation and the lumbar lordosis required for upright posture (7). In contrast, pelvic tilt and sacral slope are positional parameters that may vary as the body compensates for pain, muscle imbalance or loss of lumbar curvature (7). Disturbance of the relationship between pelvic orientation and lumbar lordosis can alter loading across the intervertebral discs, facet joints and paraspinal muscles. Abnormal sagittal alignment and paravertebral muscle degeneration have been associated with degenerative spinal conditions and impaired postural compensation (8). Changes involving the thoracolumbar junction and lumbar curvature may contribute to sagittal imbalance (9). In symptomatic LDH, pain-related guarding, reduced extension and altered pelvic orientation may perpetuate disability (8, 9).

Pelvic correction manipulation is intended to interrupt this cycle through graded soft-tissue relaxation and positional reduction selected according to symptom severity and tolerance. This approach may improve lumbar extension, promote a more favorable relationship among lumbar lordosis, pelvic tilt and sacral slope, and reduce loading of symptomatic spinal structures (7–9). It may also complement acupuncture by targeting mechanical and postural contributors that needling alone may not fully address (5, 6). The intervention could be associated with changes in inflammatory activity because LDH symptoms reflect both mechanical compression and inflammatory irritation around the herniated disc and nerve root. However, radiographic, mobility or biomarker changes should not be interpreted automatically as evidence of a causal mechanism.

Important evidence gaps remain. It is unclear whether adding a structured, alignment-directed pelvic correction protocol to adjacent needling is associated with greater functional recovery, whether any incremental benefit persists beyond treatment, and whether the combined strategy is associated with lower symptomatic recurrence or further intervention (5, 6). It is also uncertain whether improvement occurs alongside measurable changes in lumbo-pelvic sagittal parameters and lumbar extension mobility (7–9). Evaluation of these outcomes in a characterized cohort with extended follow-up may provide useful comparative-effectiveness evidence and inform subsequent randomized trials.

Therefore, this study aimed to estimate the association between adding pelvic correction manipulation to adjacent needling and disability among adults with symptomatic, imaging-confirmed LDH treated in routine care. We evaluated pain, lumbar function, meaningful ODI response, symptomatic recurrence, further LDH treatment, occupational recovery and adverse events through 24 months, while exploring changes in lumbo-pelvic sagittal alignment, lumbar extension range of motion, circulating inflammatory markers and quality of life. Because treatment selection was not randomized, all between-cohort contrasts were interpreted as associations rather than definitive causal effects.

## Methods

### Study design and setting

This was a single-center, prospective comparative cohort study with retrospective ascertainment of long-term outcomes, conducted at the Lumbocrural Pain Specialty Clinic of the Affiliated Hospital of Jiangnan University (Wuxi, China). Patients were enrolled consecutively and assessed prospectively during treatment and at scheduled follow-up; clinical events occurring up to 24 months were subsequently ascertained from medical records, structured questionnaires and telephone contact. The study was approved by the Clinical Research Ethics Committee of the Affiliated Hospital of Jiangnan University (approval no. 2019121206), and all participants gave written informed consent. Reporting follows the STROBE recommendations for cohort studies (10). Participant flow and follow-up availability are summarized in Supplementary Table 1. A completed STROBE checklist is provided in the Supplementary Material. A targeted contextual search, rather than a systematic review, was used to identify background and comparative evidence; the databases, last-search date (July 2026), language handling and reproducible search strings are reported in the Supplementary Material.

### Participants and eligibility

Eligible patients were adults aged 20–55 years with clinical features of LDH (concordant low back and radicular leg pain, with or without sensory or motor signs) confirmed by magnetic resonance imaging showing disc herniation at a level consistent with the symptomatic nerve root. A baseline VAS between 3 and 8 was required, and patients had not used analgesic or hormonal medication in the 2 weeks before assessment. Exclusion criteria were conditions requiring surgical referral (for example, high-grade spondylolisthesis, vertebral fracture, spinal infection or tumor, severe osteoporosis, marked central stenosis, or herniation causing cord or cauda equina compression); severe hepatic, renal, cardiovascular, cerebrovascular or hematological disease; severe psychiatric illness; acute infection; pregnancy or lactation; and inability to complete the assessment questionnaires.

### Exposure definition and treatment cohorts

The exposure of interest was the initial treatment strategy chosen in routine care: adjacent needling combined with pelvic correction manipulation (the combined-therapy cohort) or adjacent needling alone (the adjacent-needling cohort). Treatment was selected jointly by the treating clinician and the patient on the basis of clinical assessment and preference, and was not allocated by the investigators. The principal recorded reason for the initial choice was documented for every patient (Supplementary Table 2); clinician-identified lumbo-pelvic dysfunction and patient preference were the most common drivers, and because such factors may influence expectation, adherence and outcome, they were treated as confounders in the analysis. The primary analysis used an initial treatment-strategy estimand: participants were analyzed by the treatment they began, with an as-treated sensitivity analysis for the few who crossed over. Because “intention-to-treat” properly refers to randomized allocation, we avoid that term for this observational cohort. The handling of crossover, surgery and missing data for each outcome is set out in Supplementary Table 13.

### Interventions

#### Adjacent needling (both cohorts)

Needling was delivered by licensed acupuncturists using sterile, single-use stainless-steel filiform needles, following the STRICTA recommendations for acupuncture reporting (11). Points were selected by symptom region. For axial low back pain, the Governor Vessel point and the bilateral Jiaji (EX-B2) points at the symptomatic segment were needled perpendicularly and obliquely, respectively, to 0.8–1.0 cun. For gluteal pain, Huantiao (GB30) was needled to 2.0–2.5 cun with an adjacent oblique needle 1 cun inferior; for posterior calf pain, Weizhong (BL40) and Weiyang (BL39); and for lateral calf pain, Yanglingquan (GB34) and a point 1 cun inferior. For the adjacent-needling technique, the perpendicular needle was placed at the principal painful site and the oblique needle inserted at 45° with its tip converging on the first. At GB30, BL40 and GB34 a propagated sensation down the limb (de qi) was sought one to three times. Needles were retained for 30 min with manual stimulation at 15 min. Treatment was given once daily, 5 days per week, for ten sessions over 2 weeks.

#### Pelvic correction manipulation (combined-therapy cohort)

Patients in the combined-therapy cohort additionally received pelvic correction manipulation after needling. Because the manual component was titrated to symptom severity, its intensity was not uniform, and we report this explicitly. Pain was reassessed before each session. When the VAS was ≥5, only soft-tissue relaxation (rolling, kneading and four-finger pushing over the lumbar, gluteal and lower-limb regions at 120–150 cycles per minute for 10–15 min) was applied. When the VAS was <5, relaxation was followed by pelvic reduction techniques performed in prone (knee–chest), supine (knee–chest) and lateral (pelvic-torsion) positions, using a graded, tolerance-limited impulse repeated five times per side. Over the treatment course, 89 of 106 patients (84.0%) received pelvic reduction on at least one occasion, whereas 17 (16.0%) received relaxation only because the VAS remained ≥5 or for safety reasons; the median number of sessions containing reduction was 6 (IQR 4–8) (Supplementary Table 3). Manipulation was delivered mainly by a single experienced practitioner (88.7% of patients), and treatment fidelity was documented with a checklist in 95.3%. Because the manual component was delivered after needling, combined-therapy sessions were longer than needling-alone sessions (mean 44.2 versus 30.8 min; Supplementary Table 3); the design therefore cannot separate the specific effect of pelvic reduction from the accompanying soft-tissue relaxation, additional treatment contact time and clinician attention, an issue we return to when discussing limitations.

Adherence was high and similar between cohorts: a mean of 9.55 ± 1.08 and 9.41 ± 1.21 sessions were completed, and 95.3% and 94.1% of patients completed at least eight sessions. Rescue analgesia and unplanned physiotherapy were recorded as co-interventions (Supplementary Table 3).

### Outcomes

The prespecified primary outcome was the change in ODI, a validated 0–100 measure of low-back-related disability, from baseline to the end of treatment (12). Key secondary outcomes were the VAS for pain (0–10); the JOA score for low back pain (0–29, higher indicating better function); ODI responder status (≥30% and ≥50% improvement, referenced to the accepted minimal important change for the ODI (13)); symptomatic recurrence; and adverse events, all ascertained through 24 months. Recurrence was defined *a priori* as return or clinically important worsening of radicular pain after initial improvement (an increase of at least two VAS points with an unplanned clinical visit, renewed treatment, new analgesic prescription, injection or surgery). Exploratory outcomes were the lumbo-pelvic sagittal parameters (LL, PT, SS, PI), lumbar extension ROM, circulating IL-1β, IL-6 and TNF-α, and the four WHOQOL-BREF domains (14). Radiographic and biomarker outcomes were interpreted as mechanistic correlates rather than as evidence of a defined causal pathway.

### Radiographic and mobility measurement

Sagittal parameters were measured on standardized standing (weight-bearing) lateral lumbosacral radiographs acquired on the same equipment with a fixed source-to-image distance and standardized hip and knee position. Images were measured with dedicated software by two readers who were blinded to cohort and to clinical outcome. Lumbar extension ROM was measured with a dual-inclinometer technique (T12 and S2 spinous processes) as the mean of three maximal active trunk extensions. Reliability was quantified in a random subset of 40 examinations: intra- and inter-rater intraclass correlation coefficients were ≥0.88 for all parameters, and the minimal detectable change (MDC95) was 3.33° for lumbar lordosis and 3.05° for ROM (Supplementary Table 4), against which the observed changes were interpreted.

### Laboratory measurement

Fasting (≥8 h) peripheral venous blood was drawn before the first and after the final treatment into EDTA tubes; samples were therefore plasma. Blood was centrifuged at 1,500×*g* for 10 min at 4 °C and the plasma stored at −70 °C in single-use aliquots to avoid repeated freeze–thaw. IL-1β, IL-6 and TNF-α were quantified by double-antibody sandwich ELISA (Wuhan Boster Biological Technology Co., Ltd) in duplicate by laboratory staff blinded to cohort. Mean intra-assay coefficients of variation were 4.3–5.1% and inter-assay coefficients 6.1–6.9%; duplicate pairs with a coefficient of variation exceeding 15% were repeated (Supplementary Table 5).

### Sample size

No *a priori* sample-size calculation was performed. The sample size was determined by the number of eligible consecutive patients enrolled during the study period; we therefore emphasize the precision of estimates through 95% confidence intervals rather than reliance on statistical significance alone. This is acknowledged as a limitation of the level of evidence. To describe the resulting precision without relying on uninformative observed power, we report a minimum detectable difference. Under a simple two-group approximation, the achieved sample (about 100 patients per cohort, observed ODI standard deviation near 9.5 points) could detect a between-group difference of approximately 3.7 points with 80% power at a two-sided alpha of 0.05. This calculation is descriptive and does not fully account for weighting, repeated measures, missingness or model uncertainty. Uncommon events such as lumbar surgery, epidural injection and neurological deterioration occurred too rarely for reliable comparison, and the corresponding estimates are reported as imprecise and hypothesis-generating rather than as evidence of equivalence.

### Statistical analysis

Baseline characteristics are summarized as mean ± SD or *n* (%), with standardized mean differences (SMD) between cohorts; an absolute SMD above 0.10 was regarded as a meaningful imbalance, so that comparability was judged by SMD rather than by baseline *P* values. For longitudinal outcomes measured at multiple time points, associations were estimated with baseline-adjusted linear mixed-effects models including cohort, time, the cohort-by-time interaction, and prespecified covariates (baseline outcome value, age, sex, body-mass index, symptom duration, manual occupation, analgesic use, previous LDH, neurological deficit, herniation level and morphology, Pfirrmann degeneration grade (15), baseline sagittal parameters, treatment preference and principal practitioner). For outcomes measured at two time points, ANCOVA on the post-treatment value with the baseline value as covariate was used.

Confounding was additionally addressed with propensity-score overlap weighting, which yields exact mean balance on included covariates and estimates an effect in the population of clinical equipoise (16). Balance was assessed with weighted SMDs and a Love plot, and weighting diagnostics (effective sample size, weight distribution) were reported. Time-to-event outcomes (recurrence, additional treatment, surgery) were analyzed with Cox models, with surgery treated as a competing event in sensitivity analysis. Missing outcome data were handled by multiple imputation using chained equations (50 imputed datasets), with estimates combined by Rubin’s rules (17); complete-case, per-protocol, as-treated and worst-case (pattern-mixture) analyses were prespecified as sensitivity analyses. For the exploratory biomarker, imaging and WHOQOL families, *P* values were controlled for the false-discovery rate; exploratory outcomes are labeled as such and interpreted cautiously. Analyses were performed in R; two-sided *P* < 0.05 was considered statistically significant, and *P* values below 0.001 are reported as *P* < 0.001. Robustness of the principal associations to a single unmeasured confounder was assessed with E-values, which express the minimum association, on the risk-ratio scale, that such a confounder would need with both treatment selection and outcome, beyond the measured covariates, to move the estimate to the null (18); E-values were computed for the point estimate and for the confidence limit nearest the null. For serious adverse events with no observed cases, the upper bound of the 95% confidence interval was approximated using the rule of three (about 3/n after zero events) (19). The stability of the primary result under departures from the missing-at-random assumption was examined with a delta-adjusted (tipping-point) pattern-mixture analysis. E-values, the tipping-point analysis, number-needed-to-treat estimates, the landmark recurrence analysis and the subgroup forest plot were added during revision and are reported as post hoc or exploratory. Full model specifications, the propensity-score formula, the coding of treatment preference, the mixed-model and variance structure, the combination of overlap weights with multiple imputation, proportional-hazards diagnostics, the competing-risk treatment of surgery, and software and package versions are provided in the statistical analysis plan and analysis code in the Supplementary Material; propensity-score overlap is shown in Supplementary Figure 2. The principal practitioner was recorded but, as a post-exposure characteristic, was not included as a baseline confounder.

## Results

### Participants

**FIGURE 1:**
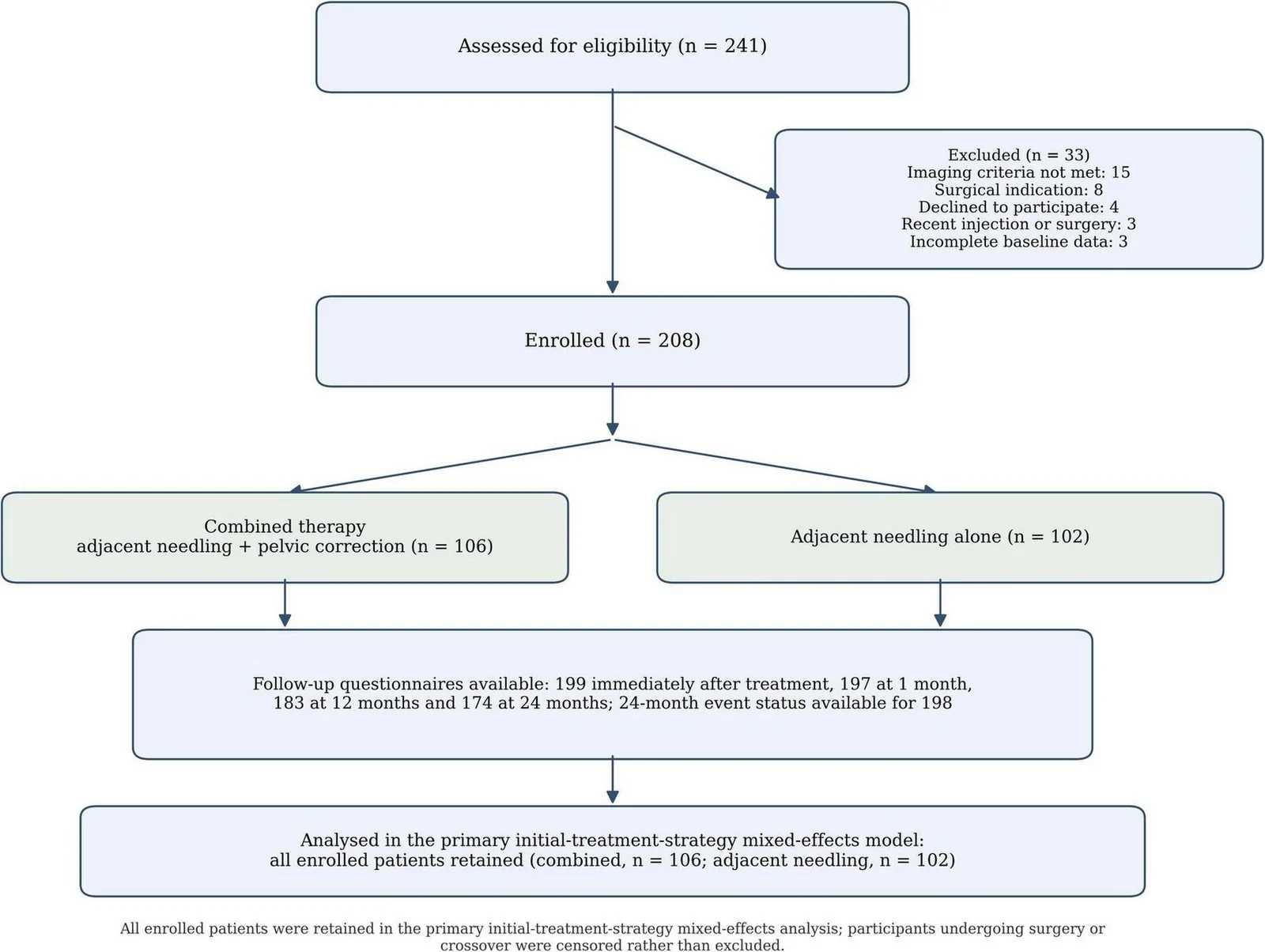
Participant flow. Flow of patients from screening through enrolment, follow-up availability and analysis. Of 241 patients screened, 208 were enrolled (combined-therapy cohort, *n* = 106; adjacent-needling cohort, *n* = 102). All enrolled patients were retained in the primary initial-treatment-strategy mixed-effects analysis; participants undergoing surgery or crossover were censored rather than excluded.

Between December 2019 and December 2020, 241 patients were screened and 208 were enrolled after 33 exclusions (Figure 1); 106 entered the combined-therapy cohort and 102 the adjacent-needling cohort. Follow-up questionnaires were available for 199 patients immediately after treatment, 197 at 1 month, 183 at 12 months and 174 at 24 months, and 24-month event status was available for 198; all 208 enrolled patients were retained in the primary mixed-effects analysis. Missingness of the 24-month ODI (16.3% overall) did not differ meaningfully between cohorts (15.1% vs. 17.6%; adjusted OR for the association of cohort with missingness 0.86, 95% CI 0.41 to 1.78; *P* = 0.681), supporting a missing-at-random assumption for the imputation model. In the delta-adjusted tipping-point analysis the direction and approximate magnitude of the primary effect were generally preserved, although statistical significance was lost when the imputed 24-month ODI in the combined-therapy cohort was made worse by about 2.6 points per missing observation relative to the missing-at-random imputation. Binary responder outcomes were additionally analyzed under multiple imputation and inverse-probability-of-observation weighting, with results materially unchanged (Supplementary Table 11).

**FIGURE 2:**
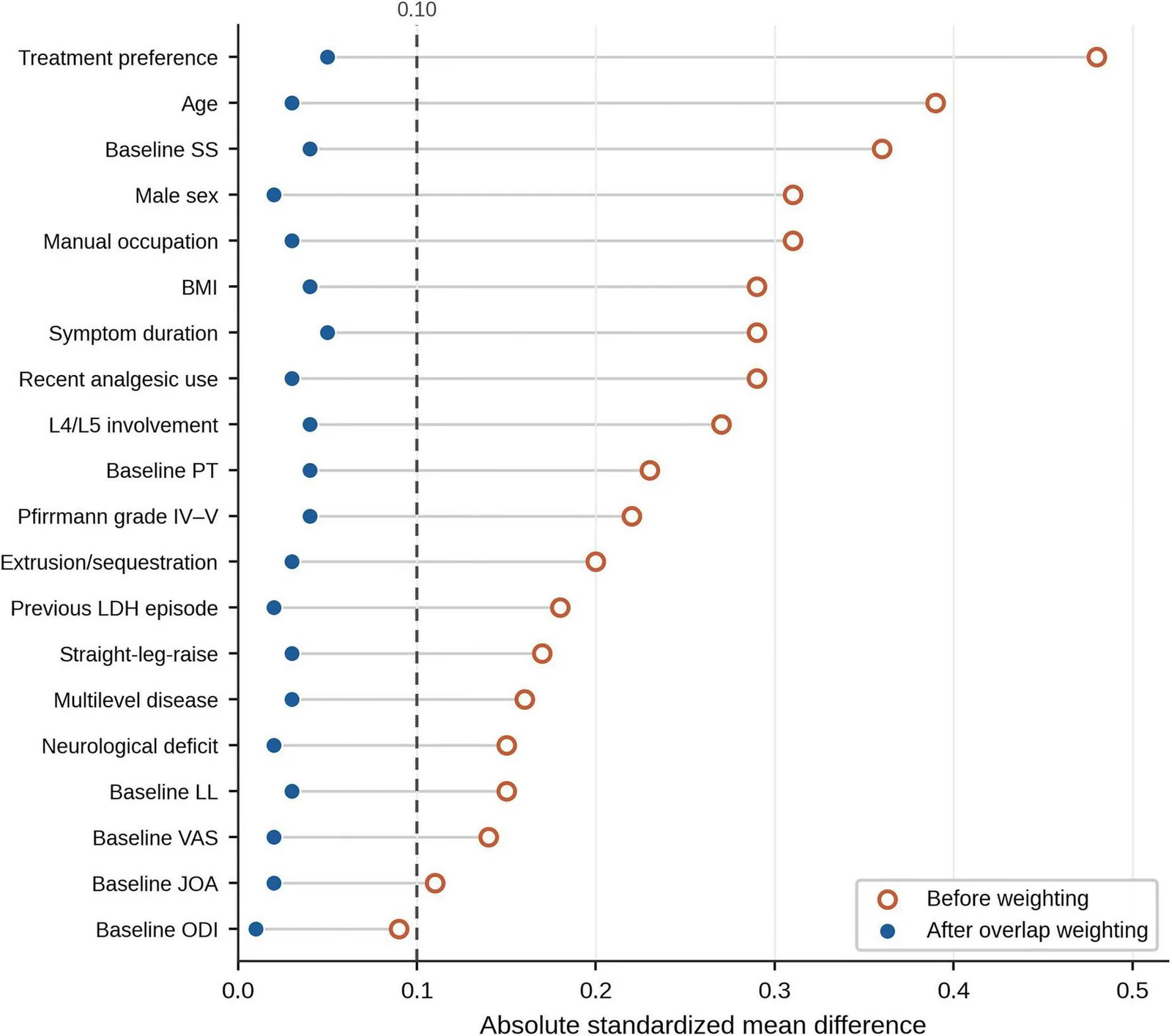
Covariate balance. Love plot of absolute standardized mean differences for baseline covariates before and after propensity-score overlap weighting. The dashed line marks the 0.10 balance threshold; after weighting all covariates were balanced (SMD ≤ 0.05).

Before adjustment, the cohorts differed on several characteristics (Table 1): patients receiving combined therapy were somewhat older (44.1 vs. 40.8 years; SMD 0.39), more often male (57.5% vs. 42.2%; SMD 0.31) and more often in heavy manual occupations (SMD 0.31), and, as expected for a preference-driven exposure, treatment preference itself was imbalanced (SMD 0.48). After overlap weighting, all covariates were closely balanced (weighted SMD ≤0.05; Figure 2, Supplementary Table 6), with effective sample sizes of 98.7 and 96.9 and no participants outside the region of common support.

**TABLE 1:** Baseline characteristics before adjustment.

Characteristic	Combined therapy (*n* = 106)	Adjacent needling (*n* = 102)	Absolute SMD
Demographic and clinical			
Age, years	44.1 ± 8.2	40.8 ± 8.6	0.39
Male sex	61 (57.5)	43 (42.2)	0.31
Body-mass index, kg/m^2^	25.1 ± 3.2	24.2 ± 3.1	0.29
Total symptom duration, months	23.7 ± 18.9	18.5 ± 16.4	0.29
Current episode duration, weeks	9.4 ± 6.7	8.6 ± 6.2	0.12
Heavy manual occupation	48 (45.3)	31 (30.4)	0.31
Current smoker	21 (19.8)	15 (14.7)	0.14
Hypertension	11 (10.4)	9 (8.8)	0.05
Diabetes mellitus	5 (4.7)	4 (3.9)	0.04
Previous LDH episode	39 (36.8)	29 (28.4)	0.18
Previous acupuncture	27 (25.5)	32 (31.4)	0.13
Analgesic use in preceding month	39 (36.8)	24 (23.5)	0.29
Positive straight-leg-raise test	72 (67.9)	61 (59.8)	0.17
Objective sensory/motor deficit	25 (23.6)	18 (17.6)	0.15
Imaging characteristics			
L4/L5 involvement	38 (35.8)	50 (49.0)	0.27
L5/S1 involvement	40 (37.7)	32 (31.4)	0.13
Multilevel involvement	28 (26.4)	20 (19.6)	0.16
Protrusion	75 (70.8)	81 (79.4)	0.20
Extrusion/sequestration	31 (29.2)	21 (20.6)	0.20
Pfirrmann grade IV–V	54 (50.9)	41 (40.2)	0.22
Baseline outcome measures			
ODI, points	29.10 ± 7.60	28.40 ± 7.50	0.09
VAS, points	6.10 ± 1.40	5.90 ± 1.50	0.14
JOA, points	13.70 ± 4.20	14.20 ± 4.50	0.11
Lumbar extension ROM, degrees	12.20 ± 3.30	12.50 ± 3.40	0.09
Lumbar lordosis, degrees	31.70 ± 3.80	31.10 ± 4.00	0.15
Pelvic tilt, degrees	20.50 ± 2.90	21.10 ± 2.30	0.23
Sacral slope, degrees	28.90 ± 5.20	27.20 ± 4.10	0.36
Pelvic incidence, degrees	49.40 ± 5.40	48.30 ± 4.60	0.22

Data are mean ± SD or *n* (%). SMD, standardized mean difference; LDH, lumbar disc herniation; ODI, Oswestry Disability Index; VAS, visual analog scale; JOA, Japanese Orthopedic Association; ROM, range of motion. An absolute SMD > 0.10 indicates a potentially meaningful between-cohort imbalance.

### Primary outcome: disability

**FIGURE 3:**
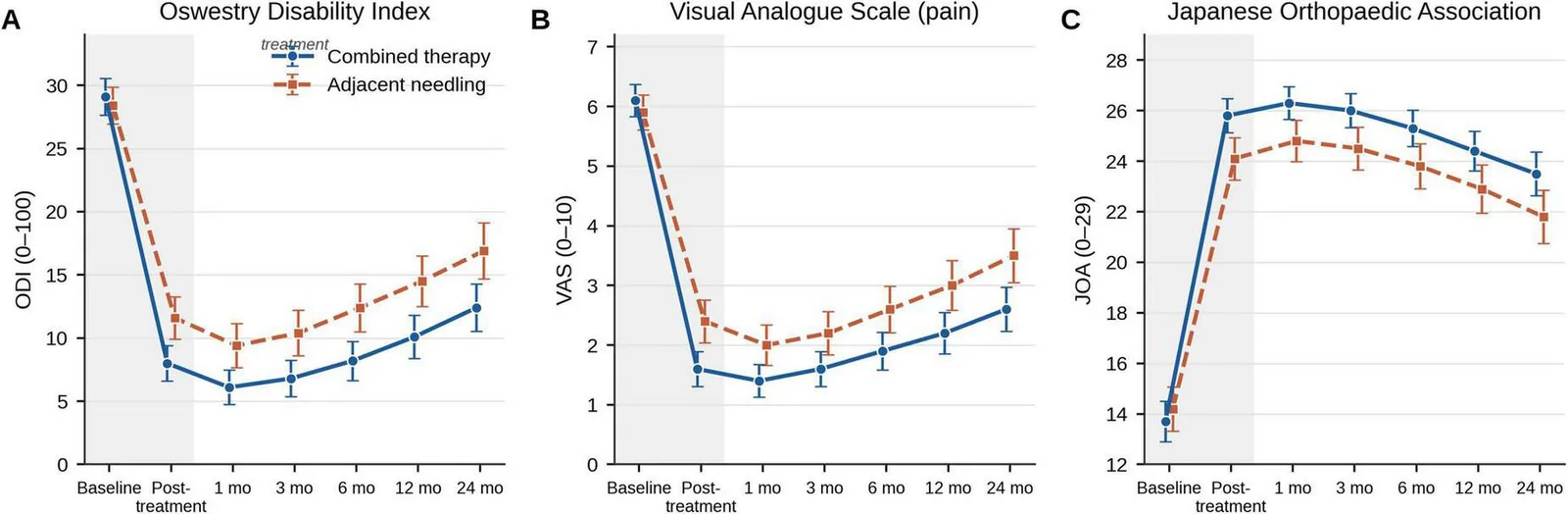
Longitudinal clinical outcomes. Mean **(A)** Oswestry Disability Index, **(B)** visual analog scale for pain and **(C)** Japanese Orthopedic Association score by cohort from baseline through 24 months. Error bars are 95% confidence intervals; the shaded band marks the ten-session treatment period. Values are from Table 2.

Both cohorts improved markedly during treatment. Mean ODI fell from 29.1 to 8.0 points in the combined-therapy cohort and from 28.4 to 11.6 in the adjacent-needling cohort, changes that exceed the accepted minimal important change of about 10 points (13). For the primary contrast, the overlap-weighted adjusted between-group difference in ODI change from baseline to the end of treatment favored combined therapy by −3.40 points (95% CI −5.40 to −1.40; *P* = 0.001), with a significant cohort-by-time interaction across the full trajectory (*P* < 0.001; Figure 3A, Tables 2, 3). The difference was maintained throughout follow-up and was numerically largest at 24 months (−4.40 points, 95% CI −7.30 to −1.50; *P* = 0.003). Because treatment was chosen in routine care rather than randomly assigned, this contrast and those that follow denote association, not causation. In the sensitivity analysis for unmeasured confounding, the E-value for the 24-month disability difference was 2.39, with an E-value of 1.57 for the confidence limit nearest the null. The point estimate therefore showed moderate robustness to a single unmeasured confounder, whereas the smaller confidence-limit value indicates that more modest residual confounding could move the interval to the null. E-values address only unmeasured confounding and do not account for selection, measurement, recall or model-specification bias.

**TABLE 2:** Longitudinal ODI, VAS and JOA outcomes by cohort.

Time point	Available *n* (C/N)	Combined therapy	Adjacent needling
Oswestry Disability Index (points)			
Baseline	106/102	29.10 ± 7.60	28.40 ± 7.50
End of treatment	102/97	8.00 ± 7.20	11.60 ± 8.40
1 month	101/96	6.10 ± 7.00	9.40 ± 8.70
3 months	99/94	6.80 ± 7.30	10.40 ± 8.90
6 months	97/92	8.20 ± 7.80	12.40 ± 9.30
12 months	94/89	10.10 ± 8.40	14.50 ± 9.70
24 months	90/84	12.40 ± 9.10	16.90 ± 10.40
Visual analogue scale for pain (points)			
Baseline	106/102	6.10 ± 1.40	5.90 ± 1.50
End of treatment	102/97	1.60 ± 1.50	2.40 ± 1.80
1 month	101/96	1.40 ± 1.40	2.00 ± 1.70
3 months	99/94	1.60 ± 1.50	2.20 ± 1.80
6 months	97/92	1.90 ± 1.60	2.60 ± 1.90
12 months	94/89	2.20 ± 1.70	3.00 ± 2.00
24 months	90/84	2.60 ± 1.80	3.50 ± 2.10
Japanese Orthopedic Association score (points)			
Baseline	106/102	13.70 ± 4.20	14.20 ± 4.50
End of treatment	102/97	25.80 ± 3.50	24.10 ± 4.20
1 month	101/96	26.30 ± 3.30	24.80 ± 4.10
3 months	99/94	26.00 ± 3.40	24.50 ± 4.20
6 months	97/92	25.30 ± 3.60	23.80 ± 4.40
12 months	94/89	24.40 ± 3.90	22.90 ± 4.60
24 months	90/84	23.50 ± 4.20	21.80 ± 4.90

Data are mean ± SD from observed data. C, combined-therapy cohort; N, adjacent-needling cohort. Adjusted between-group estimates are given in Table 3.

**TABLE 3:** Adjusted between-group estimates (combined therapy minus adjacent needling).

Outcome and time	Adjusted difference	95% CI	*P* value
Oswestry Disability Index (points)			
End of treatment	−3.40	−5.40 to −1.40	0.001
24 months	−4.40	−7.30 to −1.50	0.003
Visual analog scale (points)			
End of treatment	−0.72	−1.17 to −0.27	0.002
24 months	−0.78	−1.29 to −0.27	0.003
Japanese Orthopedic Association (points)			
End of treatment	+1.46	+0.46 to +2.46	0.004
24 months	+1.48	+0.25 to +2.71	0.019

Estimates are from baseline-adjusted linear mixed-effects models with propensity-score overlap weighting. Global cohort-by-time interaction: ODI *P* < 0.001; VAS *P* = 0.004; JOA *P* = 0.009. Full trajectory-wide estimates are provided in Supplementary material.

### Secondary clinical outcomes

**FIGURE 4:**
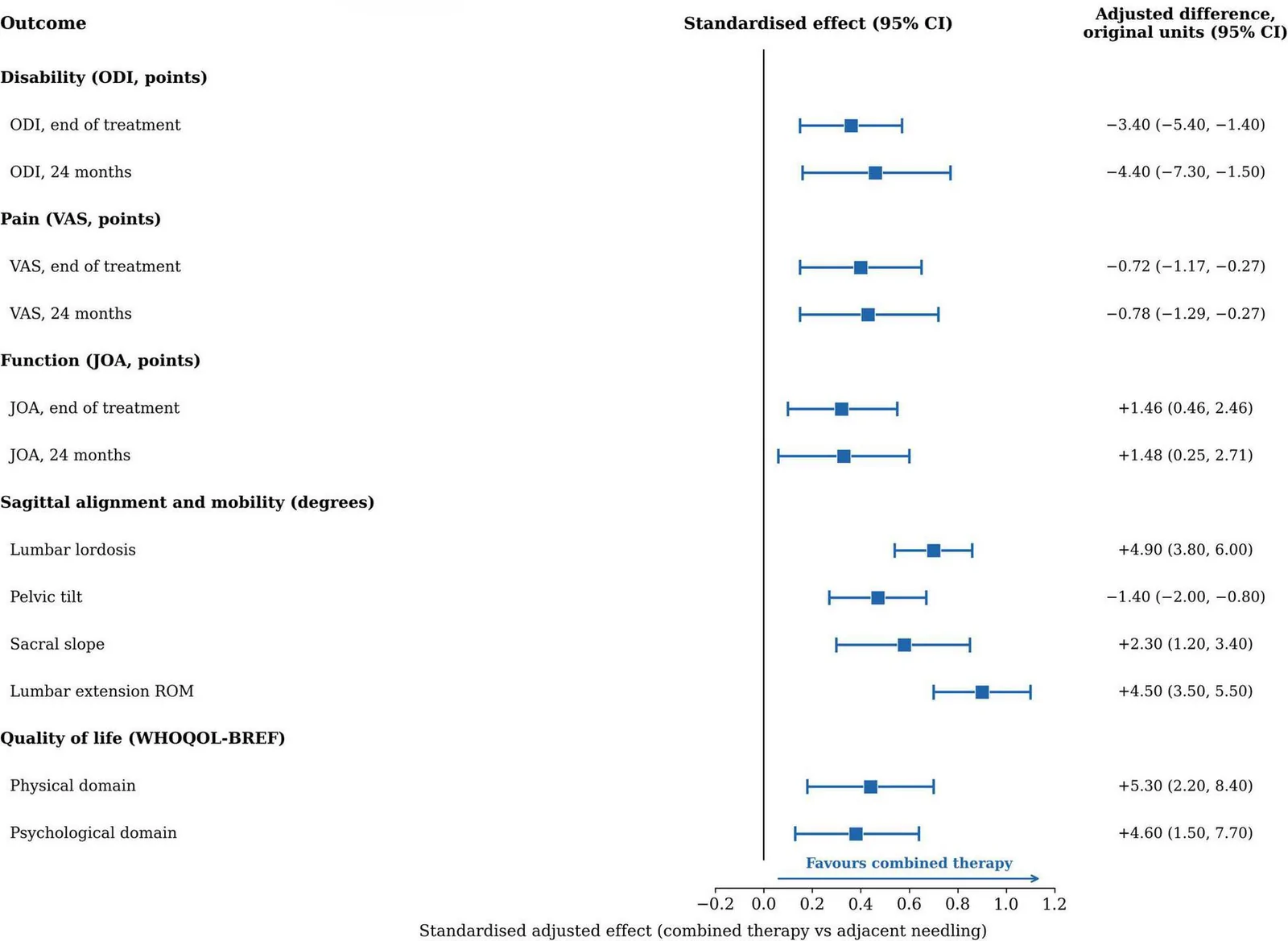
Adjusted treatment associations. Forest plot of overlap-weighted adjusted between-group differences in clinical, sagittal, mobility and quality-of-life outcomes. Standardized effects were directionally harmonized so that positive values favor combined therapy; markers and horizontal lines represent point estimates and 95% confidence intervals. The adjacent column reports adjusted between-group differences in the original measurement units, calculated as combined therapy minus adjacent needling. Negative original-unit differences favor combined therapy for ODI, VAS and pelvic tilt, whereas positive differences favor combined therapy for JOA score, lumbar lordosis, sacral slope, lumbar extension range of motion and WHOQOL-BREF scores. The standardized-effect column and the original-unit column describe the same comparisons; where their signs appear to differ, this reflects only the directional harmonization of the standardized column, in which every estimate is oriented so that values to the right favor combined therapy, whereas the original-unit column keeps each outcome’s natural sign as reported in the main text and Table 3. ODI, Oswestry Disability Index; VAS, visual analog scale; JOA, Japanese Orthopedic Association; ROM, range of motion; WHOQOL-BREF, World Health Organization Quality of Life brief version; CI, confidence interval.

Pain and lumbar function showed the same pattern. The adjusted VAS difference favored combined therapy at the end of treatment (−0.72 points, 95% CI −1.17 to −0.27) and at 24 months (−0.78, 95% CI −1.29 to −0.27), and the adjusted JOA difference favored combined therapy at the end of treatment (+1.46, 95% CI 0.46 to 2.46) and at 24 months (+1.48, 95% CI 0.25 to 2.71) (Figures 3B, C, 4 and Tables 2, 3). At 24 months, more patients receiving combined therapy reached the responder thresholds: 73.3% versus 57.1% achieved ≥30% ODI improvement (adjusted RR 1.27, 95% CI 1.04 to 1.55) and 58.9% versus 40.5% achieved ≥50% improvement (adjusted RR 1.41, 95% CI 1.06 to 1.87) (Supplementary Table 7). Occupational recovery was also more favorable: 84.1% versus 70.9% of employed patients returned to usual work by 3 months, with fewer median days of work absence (7 [IQR 3–14] vs. 12 [6–21]). To express these differences as absolute benefit, the number needed to treat derived from the adjusted responder model was about 6 for one additional patient to reach a 50% improvement in disability (95% CI 3 to 41); descriptive numbers needed for one additional observed outcome, calculated from unadjusted proportions, were about 6 for a 50% improvement, about 7 for a 30% improvement, and about 8 for return to usual work (Supplementary Table 14). These are descriptive, are not adjusted causal effects, and complement the group-level mean differences, which considered alone tend to understate the clinical relevance of the comparison.

### Recurrence and clinical events through 24 months

**FIGURE 5:**
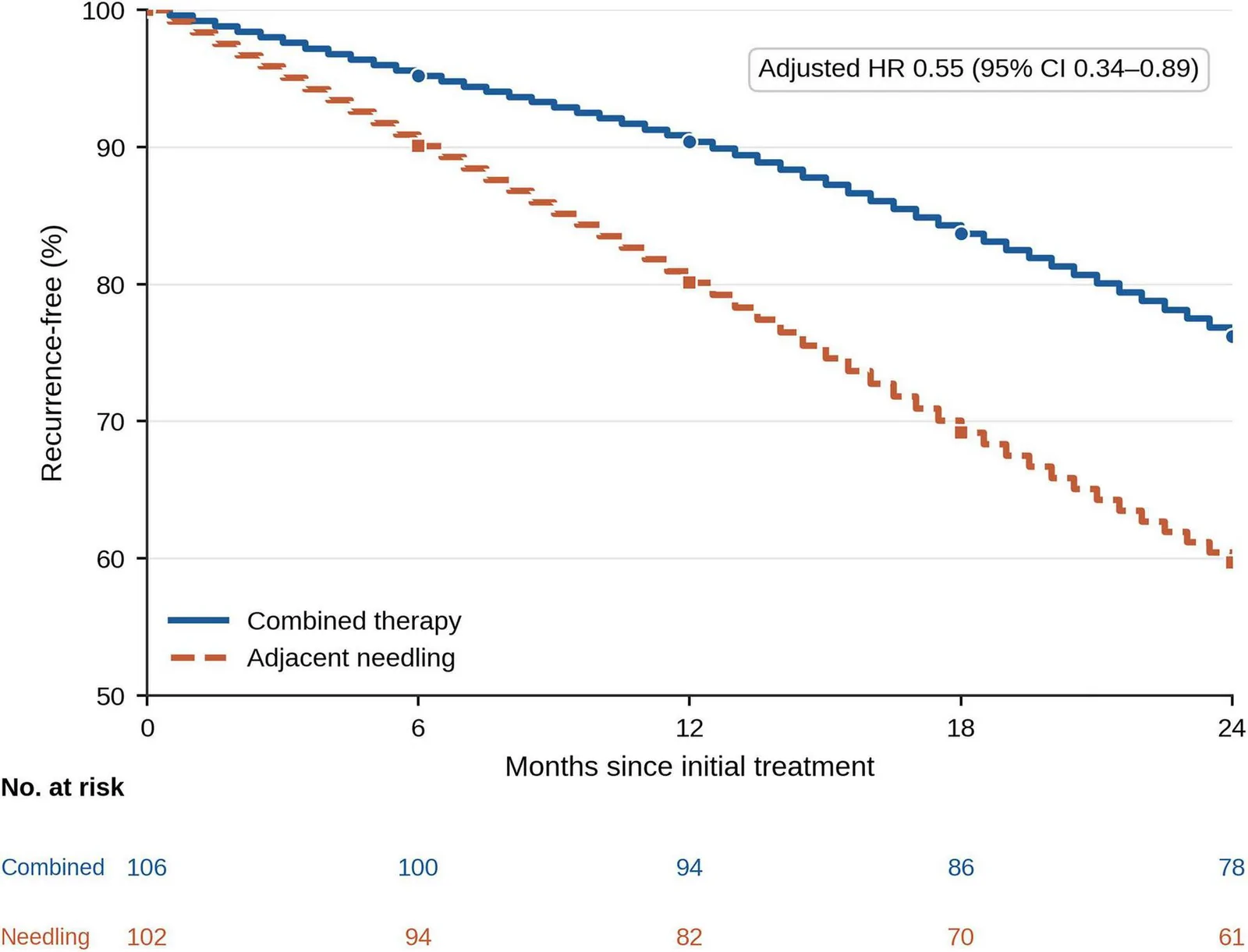
Recurrence-free survival. Adjusted recurrence-free survival by cohort over 24 months, with numbers at risk. Symptomatic recurrence was less frequent in the combined-therapy cohort (adjusted HR 0.55, 95% CI 0.34–0.89). Recurrence-free survival was estimated from treatment completion. HR, hazard ratio; CI, confidence interval.

By 24 months, symptomatic recurrence was less frequent in the combined-therapy cohort (23.6% vs. 39.2%; adjusted HR 0.55, 95% CI 0.34 to 0.89; *P* = 0.015), as was the need for additional conservative LDH treatment (20.8% vs. 38.2%; adjusted HR 0.49, 95% CI 0.29 to 0.82; *P* = 0.007) (Figure 5, Table 4). Fewer patients in the combined-therapy cohort underwent lumbar surgery (4.7% vs. 9.8%) or an epidural injection (7.5% vs. 14.7%), but these outcomes were uncommon and the confidence intervals crossed the null (surgery HR 0.47, 95% CI 0.16 to 1.38); we therefore do not interpret them as a demonstrated reduction in surgery or injection risk. Recurrence was defined as symptomatic worsening after documented initial improvement, and to give it a clear clinical time origin we performed a landmark analysis beginning at treatment completion among the patients who achieved initial improvement (97 of 106 in the combined-therapy cohort and 86 of 102 in the adjacent-needling cohort). In this landmark analysis the 24-month cumulative recurrence was 22.7% versus 38.4% (adjusted HR 0.56, 95% CI 0.34 to 0.92), consistent with the all-enrolled analysis, which is retained as a sensitivity analysis. The adjusted number needed to treat to avoid one recurrence over 24 months was about 7 (95% CI 5 to 30), and the E-value for the recurrence association was 3.04, with 1.50 for the confidence limit nearest the null.

**TABLE 4:** Clinical events through 24 months.

Event by 24 months	Combined (*n* = 106)	Needling (*n* = 102)	Adjusted effect	95% CI	*P* value
Symptomatic recurrence	25 (23.6)	40 (39.2)	HR 0.55	0.34–0.89	0.015
Additional conservative treatment	22 (20.8)	39 (38.2)	HR 0.49	0.29–0.82	0.007
Epidural/invasive nonsurgical procedure	8 (7.5)	15 (14.7)	HR 0.50	0.21–1.19	0.119
Lumbar surgery	5 (4.7)	10 (9.8)	HR 0.47	0.16–1.38	0.170
Emergency visit for radicular symptoms	9 (8.5)	17 (16.7)	HR 0.49	0.22–1.10	0.083
New/worsening neurological deficit	5 (4.7)	8 (7.8)	HR 0.59	0.19–1.82	0.360

Data are *n* (%). Effects are adjusted hazard ratios (HR) from Cox models incorporating censoring; all 208 patients contributed to time-to-event analyses. Event status was available for 101 combined-therapy and 97 adjacent-needling patients. The surgery and injection outcomes were uncommon and underpowered, and the corresponding estimates should not be interpreted as a demonstrated reduction in risk.

### Exploratory sagittal alignment and mobility

**FIGURE 6:**
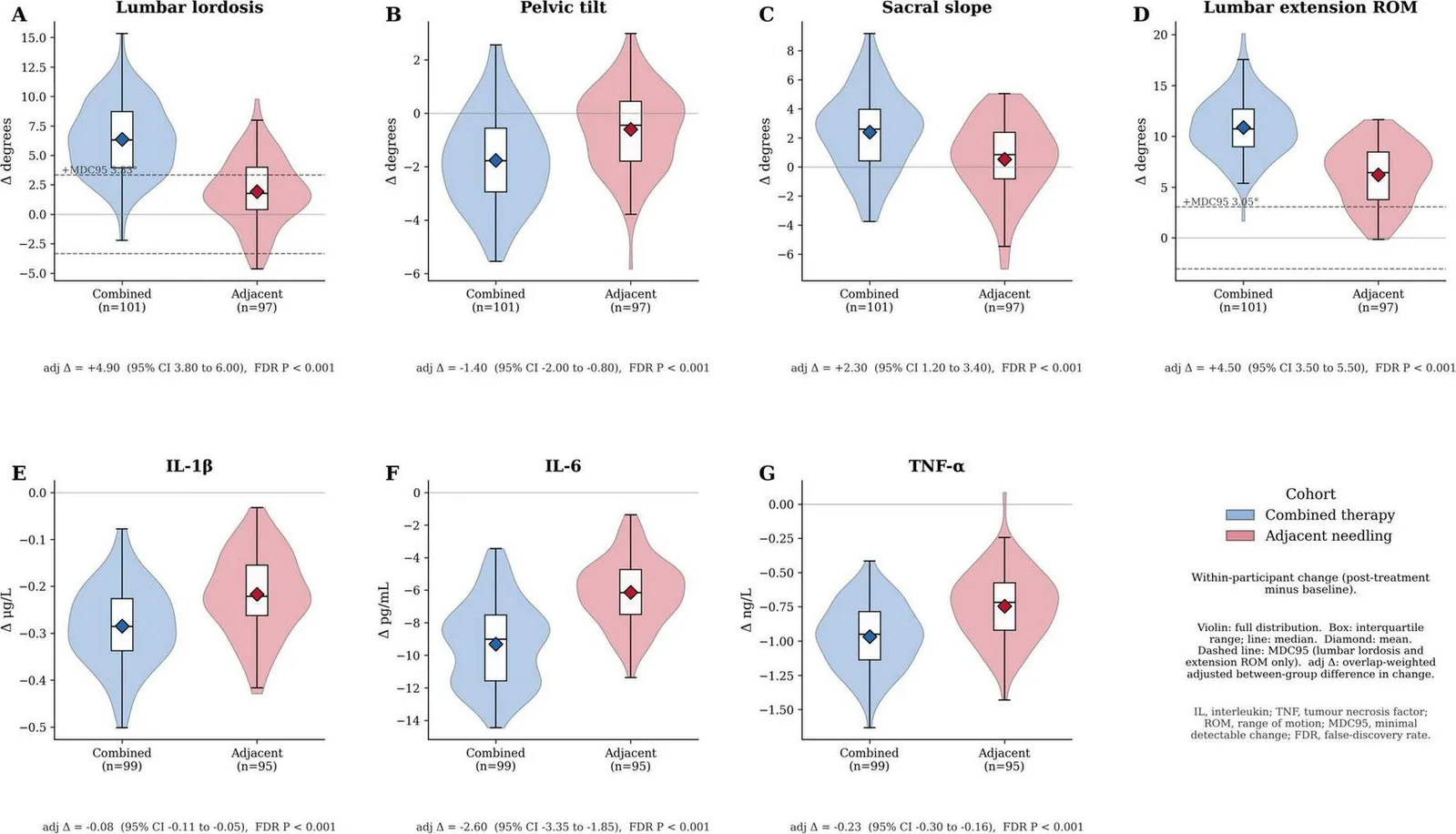
Exploratory objective outcomes. Distributions of within-participant change (post-treatment minus baseline) by cohort for lumbar lordosis, pelvic tilt, sacral slope, lumbar extension range of motion, IL-1β, IL-6 and TNF-α. Violins show the distribution, boxes the interquartile range and diamonds the mean; “adj Δ” is the overlap-weighted adjusted between-group difference in change. These outcomes were exploratory and are hypothesis-generating. Panels **A–D** show sagittal and mobility measures (lumbar lordosis, pelvic tilt, sacral slope and lumbar extension range of motion) and panels **E–G** show circulating inflammatory markers (IL-1β, IL-6 and TNF-α). Each panel is annotated with the number of paired assessments per cohort, the adjusted between-group difference with its 95% confidence interval and false-discovery-rate-adjusted P value, and, for lumbar lordosis and extension range of motion, the minimal detectable change (MDC95) as a dashed reference line. IL, interleukin; TNF, tumor necrosis factor; ROM, range of motion; MDC95, minimal detectable change at 95% confidence; FDR, false-discovery rate; adj Δ, overlap-weighted adjusted between-group difference in change.

Among patients with paired imaging, the combined-therapy cohort showed larger favorable changes in sagittal parameters: the adjusted between-group difference in change was +4.90° for lumbar lordosis (95% CI 3.80 to 6.00), −1.40° for pelvic tilt (95% CI −2.00 to −0.80) and +2.30° for sacral slope (95% CI 1.20 to 3.40); the lumbar-lordosis change exceeded the MDC95 of 3.33°. Pelvic incidence, an anatomical constant, did not change (+0.10°, 95% CI −0.50 to 0.70), as expected and consistent with the reciprocal behaviour of pelvic tilt and sacral slope (7). Lumbar extension ROM increased more in the combined-therapy cohort (+4.50°, 95% CI 3.50 to 5.50) (Figure 6, Supplementary Table 8).

### Exploratory inflammatory markers and quality of life

Circulating inflammatory markers decreased in both cohorts and more so with combined therapy, with adjusted between-group differences in change of −0.08 μg/L for IL-1β (95% CI −0.11 to −0.05), −2.60 pg/mL for IL-6 (95% CI −3.35 to −1.85) and −0.23 ng/L for TNF-α (95% CI −0.30 to −0.16) (all FDR-adjusted *P* < 0.001; Figure 6, Supplementary Table 5). Quality of life improved across all WHOQOL-BREF domains; at 24 months the between-group difference was significant for the physical (+5.30, 95% CI 2.20 to 8.40) and psychological (+4.60, 95% CI 1.50 to 7.70) domains but not for the social or environmental domains (Supplementary Table 9). Exploratory correlations linked greater gains in lumbar lordosis and ROM, and greater reductions in inflammatory markers, with greater improvement in ODI, though the coefficients were modest (| r| 0.24–0.39) and do not establish mediation (Supplementary Table 10).

### Adverse events

Adverse events were mostly minor and transient (Table 5). Any adverse event occurred in 20.8% of the combined-therapy cohort and 13.7% of the adjacent-needling cohort; the difference was not statistically clear (risk difference +7.1 percentage points, 95% CI −3.5 to +17.7; adjusted RR 1.43, 95% CI 0.79 to 2.59). The excess consisted mainly of transient post-manipulation muscular soreness (10.4% vs. 0%). No serious treatment-related adverse event, persistent neurological injury, fracture or dislocation occurred in either cohort, and withdrawal for adverse events was rare (0.9% vs. 1.0%). The appropriate conclusion is that no clear safety difference was demonstrated, not that the two strategies are proven equally safe. The post-manipulation soreness was self-limiting, localized to the treated soft tissue, resolved within 1–3 days without further treatment, and led to no withdrawal, dose reduction or serious sequela; it is an expected consequence of graded manual therapy rather than a marker of structural harm. For the serious outcomes not seen in either cohort (persistent neurological injury, fracture, dislocation and serious treatment-related events), the rule of three places the upper 95% confidence bound at approximately 2.8% in the combined-therapy cohort and 2.9% in the adjacent-needling cohort. The sample was thus too small to exclude uncommon serious harm.

**TABLE 5:** Adverse events.

Adverse event	Combined therapy (*n* = 106)	Adjacent needling (*n* = 102)
Any adverse event	22 (20.8)	14 (13.7)
Needling-site pain	8 (7.5)	7 (6.9)
Bruising or small haematoma	5 (4.7)	4 (3.9)
Transient dizziness	3 (2.8)	2 (2.0)
Vasovagal reaction	1 (0.9)	1 (1.0)
Temporary exacerbation of pain	4 (3.8)	3 (2.9)
Post-manipulation muscular soreness	11 (10.4)	0 (0.0)
Transient lower-limb numbness	2 (1.9)	1 (1.0)
Skin infection	0 (0.0)	0 (0.0)
Persistent neurological injury	0 (0.0)	0 (0.0)
Fracture or dislocation	0 (0.0)	0 (0.0)
Serious treatment-related event	0 (0.0)	0 (0.0)
Withdrawal due to adverse event	1 (0.9)	1 (1.0)

Data are *n* (%). Risk difference for any adverse event +7.1 percentage points (95% CI −3.5 to +17.7; *P* = 0.188); adjusted risk ratio 1.43 (95% CI 0.79 to 2.59). No statistically clear safety difference was demonstrated.

### Sensitivity analyses

The 24-month ODI association was stable across complete-case, overlap-weighted, conventional multivariable, per-protocol and as-treated analyses and after excluding patients who underwent surgery or crossed over (adjusted differences −4.0 to −4.7 points). Under a conservative worst-case imputation the estimate attenuated to −3.10 points (95% CI −6.20 to 0.00; *P* = 0.051), and a pattern-mixture analysis assuming missing outcomes were two points worse gave −3.70 points (95% CI −6.70 to −0.70) (Supplementary Table 11). The recurrence association was likewise robust, including when surgery was treated as a competing event (Supplementary Table 11). showed broadly consistent associations with no significant interactions (all interaction *P* > 0.05; Supplementary Table 12). Exploratory subgroup estimates with their interaction tests are displayed as a forest plot in Supplementary Figure 1, and the full quantitative sensitivity analyses, including the competing-risk treatment of surgery and the tipping-point results, are tabulated in Supplementary Table 11.

## Discussion

In this prospective comparative cohort of adults with symptomatic, imaging-confirmed lumbar disc herniation (LDH), adding pelvic correction manipulation to adjacent needling was associated with more favorable outcomes than adjacent needling alone. Combined therapy was associated with greater improvement in disability, pain and lumbar function at treatment completion, and the differences persisted through 24 months. It was also associated with higher ODI responder rates, earlier return to usual work, fewer symptomatic recurrences and less additional conservative treatment. Exploratory analyses showed concordant changes in lumbar lordosis, pelvic tilt, sacral slope, extension mobility, inflammatory markers and physical and psychological quality of life. Pelvic incidence remained unchanged, as expected for a stable morphological parameter (7). These findings suggest a potentially useful incremental effect, but the non-randomized treatment allocation prevents causal inference.

The primary ODI association should be interpreted in context. Both cohorts improved by more than the approximately 10-point individual-level minimal important change (13), indicating that adjacent needling alone was associated with substantial recovery. The adjusted between-group difference of approximately 3–4 points was smaller than that threshold and therefore represents a modest mean incremental benefit rather than a large effect. Nevertheless, a group-level contrast and an individual responder threshold answer different questions. The persistence of the difference across follow-up, together with higher proportions achieving at least 30% and 50% ODI improvement, lower recurrence and more favorable occupational recovery, provides a coherent pattern that is more clinically informative than any isolated estimate. The findings should therefore be viewed as evidence of modest but potentially meaningful comparative benefit rather than proof of a major treatment effect.

The clinical trajectory was also informative. Improvement occurred rapidly during the 2-week treatment course, followed by partial attenuation over 24 months in both groups. This pattern is plausible in LDH, a condition that may recur because of ongoing disc degeneration, occupational loading, deconditioning and renewed inflammatory irritation (1, 8). Despite this attenuation, between-cohort separation remained evident, suggesting that the association was not confined to immediate post-treatment relief. The lower incidence of symptomatic recurrence and additional conservative treatment strengthens the suggestion of greater durability. In contrast, surgery, epidural procedures and neurological deterioration were uncommon, and their confidence intervals were wide. These event estimates should not be interpreted as evidence that combined therapy reduces the need for surgery, which remains appropriate for selected patients with progressive neurological compromise or other clear indications (2).

Our results are consistent with and extend existing clinical evidence. Randomized evidence supports acupuncture for chronic sciatica caused by herniated disc (5). A four-arm randomized trial further reported that acupuncture combined with manipulation improved pain and JOA scores more than acupuncture, manipulation or traction alone over 3 months (6). The present study evaluated a specifically defined, symptom-guided pelvic correction protocol and extended observation to 24 months, including recurrence, additional treatment and occupational outcomes. It also incorporated sagittal measurements, mobility testing and inflammatory biomarkers as exploratory correlates. Differences across studies may reflect variation in patient selection, techniques, outcomes and follow-up. The routine-care design increased clinical heterogeneity but also susceptibility to confounding. The direction of benefit is consistent with disease-specific guidance and recent trials: the 2024 evidence-based Chinese-medicine guideline for lumbar disc herniation supports acupuncture and manipulative therapy within conservative care, and recent randomized studies of acupuncture or manual therapy combined with other conservative treatments report improved function and pain in this population, though several have short follow-up or limited blinding (4, 6, 22, 23). The 2023 World Health Organization guideline provides broader low-back-pain context rather than herniation-specific direction (3).

Several mechanisms could plausibly contribute to the observed associations, although the present study cannot establish them. Disc-related radicular symptoms arise from both mechanical deformation and inflammatory irritation of the affected nerve root. Herniated disc material may stimulate cytokine responses involving IL-1β, IL-6 and TNF-α, contributing to nociceptor sensitisation, neural oedema and disc degeneration (20, 21, 24, 25). Adjacent needling may modulate pain processing, muscle tone and inflammatory activity (5), whereas soft-tissue relaxation and graded pelvic reduction may additionally reduce protective spasm, improve movement and lessen mechanically provoked neural irritation. The greater reductions in circulating inflammatory markers are compatible with this interpretation, but circulating concentrations are nonspecific and do not measure local disc or nerve-root inflammation. They are associated signals rather than evidence of a defined pathway. Peripheral cytokine concentrations reflect systemic inflammatory activity and cannot be localized to the herniated disc or the affected nerve root, so their decline is best read as a correlate of clinical improvement rather than a measured mechanism of it, even though local disc and periradicular tissue is a recognized source of these mediators (20, 21, 24, 25).

The sagittal and mobility findings provide a complementary biomechanical hypothesis. The observed increase in lumbar lordosis and sacral slope, reduction in pelvic tilt and improvement in extension range of motion may reflect reduced guarding and more favorable lumbo-pelvic movement rather than structural alteration of the pelvis. Abnormal sagittal alignment and paraspinal degeneration have been associated with impaired compensation in degenerative spinal disorders (8, 9). The change in lumbar lordosis exceeded the minimal detectable change, making measurement error alone unlikely. However, pain relief may itself permit a more natural posture and greater extension. The modest correlations with ODI do not establish mediation; these changes may have occurred in parallel. Because the radiographic and mobility measures were obtained after symptoms had eased, an apparent improvement in alignment could be a consequence of pain relief rather than its cause; the exploratory correlations are modest and are compatible with either direction of effect.

The recurrence and occupational findings may be especially relevant to clinical decision-making. For working-age adults, the value of conservative treatment includes sustained function, avoidance of repeated care and return to normal activity, not merely short-term pain reduction (1). Combined therapy was associated with fewer recurrences and additional treatments, faster return to usual work and a higher proportion maintaining usual work at 24 months. These outcomes suggest relevance beyond questionnaire scores. However, recurrence and employment are influenced by occupation, workplace accommodation, socioeconomic conditions and expectations; the differences cannot be attributed exclusively to manipulation. Occupational recovery is shaped by job demands, workplace accommodation, income protection and local labor-market conditions as much as by symptom relief, and these factors were only partly captured; the return-to-work contrast is therefore best read as a patient-relevant association rather than a direct treatment effect.

Clinically, these findings support further evaluation of adding pelvic correction manipulation in a prospectively registered, multicentre, attention-matched randomized trial, rather than establishing its routine clinical effectiveness, and any current use would be for selected adults whose LDH is accompanied by restricted extension for selected adults whose LDH is accompanied by restricted extension, postural guarding or suspected lumbo-pelvic dysfunction. The symptom-guided protocol deferred pelvic reduction when pain remained severe, and some participants received relaxation only. This may improve tolerability but does not justify universal application or replacement of established care pathways. Patients with progressive weakness, cauda equina features, instability, fracture, infection, tumor or other surgical indications require prompt specialist assessment (2). Observed events were mainly transient soreness and needling-related events and no serious treatment-related complications. However, the cohort was too small to detect rare harms, so equivalence of safety between strategies cannot be concluded. Because care was delivered at a single center and most manipulation was performed by one experienced practitioner, the effect estimates may not transfer directly to other settings or to less experienced operators, and external validation across multiple centers and therapists is needed.

This study has several strengths. It included 208 consecutively enrolled patients with imaging-confirmed LDH, a prespecified primary outcome and 24-month ascertainment of clinically meaningful events. Analyses combined baseline-adjusted mixed-effects models with propensity-score overlap weighting, achieving close balance in measured covariates. Missing outcomes were addressed by multiple imputation, and findings were examined across several sensitivity analyses. Treatment adherence, crossover, co-interventions and fidelity were documented. Imaging and biomarker assessments were blinded and quality controlled. Responder, recurrence and return-to-work outcomes improved clinical interpretability.

The limitations are substantial. Treatment was selected jointly by clinicians and patients rather than randomly assigned, creating potential confounding by indication. Although overlap weighting balanced measured characteristics, unmeasured differences in expectations, motivation, psychosocial status, self-management, clinician judgment and prior treatment experience may remain. Participants and therapists were not blinded, and combined therapy involved longer sessions and greater contact; attention and expectation may therefore have influenced patient-reported outcomes. The intervention was heterogeneous and delivered predominantly by one practitioner. The study cannot isolate pelvic reduction from relaxation, additional contact time or practitioner effects. Because the 17 patients who received soft-tissue relaxation only were selected by persistent pain severity and were too few for a stable comparison, we do not attempt a component-specific estimate; separating the contribution of pelvic reduction from relaxation, treatment contact time and clinician attention will require a randomized, attention-matched comparison.

Generalisability is also limited by the single-center setting and restriction to adults aged 20–55 years. Some long-term outcomes were ascertained retrospectively, and questionnaire data were missing for a proportion of participants, although multiple imputation and sensitivity analyses were used. No *a priori* sample-size calculation was performed, and uncommon outcomes such as surgery and neurological deterioration were underpowered. The imaging and biomarker analyses were exploratory, involved participants with paired measurements and were not supported by formal mediation analysis. Multiplicity control does not eliminate chance findings. Radiographs may reflect pain-related positioning, and circulating cytokines may not represent local tissue biology (7–9, 20, 21, 24, 25). Retrospective ascertainment of some long-term events through telephone contact and questionnaires introduces potential recall bias; we limited this by cross-checking self-reports against medical records for treatment, injection and surgery, but misclassification of milder recurrences cannot be excluded. Rescue analgesia and unplanned physiotherapy were recorded (Supplementary Table 3), but home exercise, physical activity and other self-management during follow-up were not captured in detail, and unmeasured differences in these behaviors could influence the recurrence and durability findings.

Future research should use a prospectively registered, multicenter randomized design with concealed allocation, blinded assessors and an attention-matched comparator. The protocol should be standardized while retaining safety-based modifications, with fidelity assessed across practitioners. Follow-up of 12–24 months should include disability response, recurrence, healthcare use, return to work, surgery and adverse events, repeated imaging and biomarkers, and formal mediation analyses could clarify who benefits and whether biomechanical or inflammatory changes precede clinical improvement. Economic evaluation is also warranted.

## Conclusion

In this prospective comparative cohort, adding pelvic correction manipulation to adjacent needling was associated with greater short- and longer-term improvement in disability, pain and lumbar function and with lower symptomatic recurrence. Exploratory changes in lumbo-pelvic alignment, lumbar mobility, circulating inflammatory markers and quality of life were also observed. These findings are consistent with evidence supporting acupuncture for disc-related sciatica and the short-term use of acupuncture combined with manipulation (5, 6). However, the incremental mean disability benefit was modest, and non-randomized treatment selection, lack of participant and therapist blinding, intervention heterogeneity and residual confounding limit causal interpretation. Confirmation in a prospectively assessor-blinded randomized trial is required.

## Data Availability

The original contributions presented in the study are included in the article/Supplementary material, further inquiries can be directed to the corresponding author.
