# Supplementary material for: Clinical outcomes associated with adding pelvic correction manipulation to adjacent needling for lumbar disc herniation: a prospective comparative cohort study
Source: Front Med (Lausanne). 2026 Aug 24;13:1936013. doi: 10.3389/fmed.2026.1936013 (PMC13547943; doi:10.3389/fmed.2026.1936013)
Supplement: Supplementary file 1 [file Data_Sheet_1.docx]

**Supplementary Material**

**Table S1.** Participant flow and follow-up availability

| **Study stage** | **Combined therapy** | **Adjacent needling** | **Total** |
| --- | --- | --- | --- |
| Patients screened | — | — | 241 |
| Imaging/clinical criteria not met | — | — | 15 |
| Immediate surgical indication | — | — | 8 |
| Declined participation | — | — | 4 |
| Recent injection or lumbar surgery | — | — | 3 |
| Incomplete baseline assessment | — | — | 3 |
| Enrolled | 106 | 102 | 208 |
| Completed ≥8 sessions | 101 | 96 | 197 |
| End-of-treatment assessment | 102 | 97 | 199 |
| 1-month questionnaire | 101 | 96 | 197 |
| 3-month questionnaire | 99 | 94 | 193 |
| 6-month questionnaire | 97 | 92 | 189 |
| 12-month questionnaire | 94 | 89 | 183 |
| 24-month questionnaire | 90 | 84 | 174 |
| 24-month recurrence/surgery status | 101 | 97 | 198 |
| Included in mixed-effects analysis | 106 | 102 | 208 |
| Complete-case 24-month ODI analysis | 90 | 84 | 174 |

**Reasons for missing 24-month questionnaire** (combined/needling/total): unable to contact 7/8/15; moved outside region 3/3/6; withdrew from further questionnaires 2/2/4; serious unrelated illness 2/2/4; death unrelated to LDH or treatment 1/1/2; administrative loss 1/2/3. Total 16/18/34. All 208 patients were retained in the analytical cohort; patients who underwent surgery or changed treatment were censored, not deleted.

**Table S2.** Recorded principal reason for initial treatment selection

| **Principal reason for initial treatment choice** | **Combined therapy (n = 106)** | **Adjacent needling (n = 102)** |
| --- | --- | --- |
| Clinician-identified lumbo-pelvic dysfunction/asymmetry | 40 (37.7) | 0 |
| Patient preference for combined treatment | 29 (27.4) | 0 |
| Inadequate response to previous needling | 18 (17.0) | 0 |
| Previous favourable response to manipulation | 9 (8.5) | 0 |
| High occupational functional demand | 10 (9.4) | 0 |
| Patient preference for needling alone | 0 | 39 (38.2) |
| Concern about or refusal of manipulation | 0 | 20 (19.6) |
| Acute symptom irritability influencing choice | 0 | 17 (16.7) |
| Previous favourable response to acupuncture | 0 | 14 (13.7) |
| Manipulation unavailable (scheduling) | 0 | 12 (11.8) |

Data are n (%). Treatment preference and clinician selection were treated as confounders because they may influence expectation, adherence and outcome.

**Table S3.** Treatment exposure, adherence and fidelity

| **Variable** | **Combined therapy (n = 106)** | **Adjacent needling (n = 102)** |
| --- | --- | --- |
| Sessions completed, mean ± SD | 9.55 ± 1.08 | 9.41 ± 1.21 |
| Sessions completed, median (IQR) | 10 (9–10) | 10 (9–10) |
| Completed all 10 sessions | 88 (83.0) | 82 (80.4) |
| Completed ≥8 sessions | 101 (95.3) | 96 (94.1) |
| Completed <8 sessions | 5 (4.7) | 6 (5.9) |
| Mean session duration, minutes | 44.2 ± 6.1 | 30.8 ± 4.0 |
| Rescue analgesic during treatment | 13 (12.3) | 19 (18.6) |
| Unplanned physiotherapy | 4 (3.8) | 6 (5.9) |
| Major protocol deviation | 4 (3.8) | 5 (4.9) |
| Crossed to alternative treatment | 2 (1.9) | 4 (3.9) |
| Stopped for adverse effects | 1 (0.9) | 1 (1.0) |

**Combined-treatment components** (pain reassessed before each session): received relaxation at least once 106 (100%); received pelvic reduction at least once 89 (84.0%); relaxation only because VAS remained ≥5 or for safety 17 (16.0%); sessions containing pelvic reduction, median (IQR) 6 (4–8); prone knee–chest used 86 (81.1%); supine knee–chest 83 (78.3%); lateral pelvic torsion 79 (74.5%); treated principally by the same practitioner 94 (88.7%); fidelity checklist completed 101 (95.3%).

**Table S4.** Measurement reliability of radiographic and ROM outcomes

| **Measurement** | **Intra-rater ICC** | **Inter-rater ICC** | **SEM** | **MDC95** |
| --- | --- | --- | --- | --- |
| Lumbar lordosis | 0.94 | 0.91 | 1.20° | 3.33° |
| Pelvic tilt | 0.92 | 0.89 | 0.80° | 2.22° |
| Sacral slope | 0.93 | 0.90 | 1.10° | 3.05° |
| Pelvic incidence | 0.96 | 0.94 | 0.70° | 1.94° |
| Lumbar extension ROM | 0.91 | 0.88 | 1.10° | 3.05° |

Forty imaging examinations and 40 ROM assessments were re-measured. Both readers were blinded to cohort and to clinical outcomes (40/40); unresolved discrepancies 0; measurements repeated for positioning concern 3. ICC, intraclass correlation coefficient; SEM, standard error of measurement; MDC95, minimal detectable change at 95% confidence.

**Table S5.** Exploratory inflammatory-marker outcomes and assay quality

| **Marker** | **Comb. baseline** | **Comb. post** | **Ctrl baseline** | **Ctrl post** | **Adj. Δ (95% CI)** |
| --- | --- | --- | --- | --- | --- |
| IL-1β, μg/L | 0.67 ± 0.12 | 0.38 ± 0.10 | 0.68 ± 0.13 | 0.47 ± 0.11 | −0.08 (−0.11, −0.05) |
| IL-6, pg/mL | 23.40 ± 2.40 | 14.20 ± 2.60 | 23.10 ± 2.50 | 16.90 ± 2.80 | −2.60 (−3.35, −1.85) |
| TNF-α, ng/L | 2.31 ± 0.28 | 1.34 ± 0.25 | 2.29 ± 0.29 | 1.58 ± 0.27 | −0.23 (−0.30, −0.16) |

Paired plasma samples available from 99 combined-therapy and 95 adjacent-needling patients; all FDR-adjusted P < 0.001. **Assay quality** (IL-1β/IL-6/TNF-α): tested in duplicate 194/194 each; mean intra-assay CV 4.8%/4.3%/5.1%; mean inter-assay CV 6.6%/6.1%/6.9%; duplicate pairs with CV > 15% repeated 3/2/4; laboratory staff blinded to cohort 194/194. FDR, false-discovery rate; CV, coefficient of variation.

**Table S6.** Propensity-score covariate balance and weighting diagnostics

| **Covariate** | **Unweighted SMD** | **Overlap-weighted SMD** |
| --- | --- | --- |
| Age | 0.39 | 0.03 |
| Sex | 0.31 | 0.02 |
| BMI | 0.29 | 0.04 |
| Symptom duration | 0.29 | 0.05 |
| Manual occupation | 0.31 | 0.03 |
| Recent analgesic use | 0.29 | 0.03 |
| Previous LDH episode | 0.18 | 0.02 |
| Straight-leg-raise test | 0.17 | 0.03 |
| Neurological deficit | 0.15 | 0.02 |
| L4/L5 involvement | 0.27 | 0.04 |
| Multilevel disease | 0.16 | 0.03 |
| Extrusion/sequestration | 0.20 | 0.03 |
| Pfirrmann grade IV–V | 0.22 | 0.04 |
| Baseline ODI | 0.09 | 0.01 |
| Baseline VAS | 0.14 | 0.02 |
| Baseline JOA | 0.11 | 0.02 |
| Baseline LL | 0.15 | 0.03 |
| Baseline PT | 0.23 | 0.04 |
| Baseline SS | 0.36 | 0.04 |
| Treatment preference | 0.48 | 0.05 |

**Weighting diagnostics** — original sample size 106/102; effective weighted sample size 98.7/96.9; mean standardized weight 1.00/1.00; maximum 1.73/1.69; minimum 0.21/0.24; participants outside common support 0/0; weighted covariates with SMD > 0.10: 0. SMD, standardized mean difference; LL, lumbar lordosis; PT, pelvic tilt; SS, sacral slope.

**Table S7.** 24-month responder and work outcomes

| **Outcome** | **Combined (n = 90)** | **Needling (n = 84)** | **Effect** | **95% CI** | **P value** |
| --- | --- | --- | --- | --- | --- |
| ≥30% ODI improvement | 66 (73.3) | 48 (57.1) | RR 1.27 | 1.04–1.55 | 0.019 |
| ≥50% ODI improvement | 53 (58.9) | 34 (40.5) | RR 1.41 | 1.06–1.87 | 0.018 |
| VAS improvement ≥2 points | 70 (77.8) | 55 (65.5) | RR 1.17 | 0.99–1.38 | 0.066 |
| VAS improvement ≥50% | 62 (68.9) | 44 (52.4) | RR 1.28 | 1.02–1.60 | 0.034 |
| JOA improvement ≥5 points | 64 (71.1) | 50 (59.5) | RR 1.17 | 0.96–1.43 | 0.119 |
| Condition ‘much/very much better’ | 61 (67.8) | 42 (50.0) | RR 1.31 | 1.03–1.68 | 0.029 |

Data are n (%) among questionnaire responders; effects are adjusted relative risks (RR). **Work outcomes** (employed at baseline 88 combined / 86 needling): returned to usual work by 3 months 74/88 (84.1%) vs 61/86 (70.9%); median days absent in first 3 months 7 (IQR 3–14) vs 12 (6–21); sustained usual work at 24 months 68/88 (77.3%) vs 55/86 (64.0%); changed work because of LDH 8/88 (9.1%) vs 15/86 (17.4%).

**Table S8.** Exploratory lumbo-pelvic and range-of-motion outcomes

| **Outcome** | **Comb. baseline** | **Comb. post** | **Ctrl baseline** | **Ctrl post** | **Adj. Δ (95% CI)** |
| --- | --- | --- | --- | --- | --- |
| LL, deg | 31.70 ± 3.80 | 38.10 ± 4.80 | 31.10 ± 4.00 | 32.90 ± 4.40 | +4.90 (3.80, 6.00) |
| PT, deg | 20.50 ± 2.90 | 18.40 ± 2.20 | 21.10 ± 2.30 | 20.50 ± 2.40 | −1.40 (−2.00, −0.80) |
| SS, deg | 28.90 ± 5.20 | 31.00 ± 5.00 | 27.20 ± 4.10 | 27.80 ± 4.20 | +2.30 (1.20, 3.40) |
| PI, deg | 49.40 ± 5.40 | 49.40 ± 5.30 | 48.30 ± 4.60 | 48.30 ± 4.60 | +0.10 (−0.50, 0.70) |
| ROM, deg | 12.20 ± 3.30 | 23.70 ± 4.40 | 12.50 ± 3.40 | 18.90 ± 4.20 | +4.50 (3.50, 5.50) |

Paired imaging available for 101 combined-therapy and 97 adjacent-needling patients. Adjusted Δ is the overlap-weighted between-group difference in change. **Three-month ROM** (baseline / post / 3 months): combined 12.20 / 23.70 / 22.80; needling 12.50 / 18.90 / 18.30 degrees. LL, lumbar lordosis; PT, pelvic tilt; SS, sacral slope; PI, pelvic incidence; ROM, lumbar extension range of motion.

**Table S9.** Exploratory WHOQOL-BREF domain scores over time

| **Domain / time** | **Baseline** | **Post** | **6 mo** | **12 mo** | **24 mo** |
| --- | --- | --- | --- | --- | --- |
| **Physical health (combined / needling)** | | | | | |
| Combined | 52.60 | 77.50 | 75.40 | 72.80 | 69.80 |
| Needling | 53.20 | 69.40 | 68.70 | 66.40 | 63.80 |
| **Psychological (combined / needling)** | | | | | |
| Combined | 54.00 | 75.60 | 73.50 | 71.20 | 68.70 |
| Needling | 54.80 | 69.80 | 68.10 | 65.90 | 63.50 |
| **Social relationships (combined / needling)** | | | | | |
| Combined | 58.00 | 73.80 | 72.00 | 69.80 | 67.20 |
| Needling | 58.90 | 70.10 | 68.80 | 66.40 | 63.90 |
| **Environmental (combined / needling)** | | | | | |
| Combined | 56.90 | 71.70 | 70.20 | 68.50 | 66.80 |
| Needling | 57.40 | 68.50 | 67.00 | 65.20 | 63.70 |

Data are mean domain scores (0–100). **Adjusted 24-month between-group differences (95% CI; FDR-adjusted P):** physical +5.30 (2.20, 8.40; 0.003); psychological +4.60 (1.50, 7.70; 0.007); social +2.80 (−0.30, 5.90; 0.084); environmental +2.70 (−0.20, 5.60; 0.079).

**Table S10.** Exploratory correlations between changes in objective outcomes and disability

| **Association** | **r** | **95% CI** | **FDR P** |
| --- | --- | --- | --- |
| Increase in LL vs decrease in ODI | −0.34 | −0.46 to −0.20 | <0.001 |
| Decrease in PT vs decrease in ODI | +0.28 | +0.14 to +0.41 | 0.001 |
| Increase in SS vs decrease in ODI | −0.24 | −0.38 to −0.10 | 0.004 |
| Increase in ROM vs decrease in ODI | −0.39 | −0.51 to −0.26 | <0.001 |
| Reduction in IL-1β vs decrease in ODI | +0.25 | +0.11 to +0.38 | 0.004 |
| Reduction in IL-6 vs decrease in ODI | +0.29 | +0.15 to +0.42 | 0.001 |
| Reduction in TNF-α vs decrease in ODI | +0.27 | +0.13 to +0.40 | 0.002 |
| Reduction in IL-6 vs reduction in VAS | +0.31 | +0.17 to +0.44 | <0.001 |

Changes coded as post-treatment minus baseline. These are associations only and do not establish mediation or a biological mechanism. LL, lumbar lordosis; PT, pelvic tilt; SS, sacral slope; ROM, range of motion; FDR, false-discovery rate.

**Table S11.** Missing-data and sensitivity analyses

| **24-month ODI analysis** | **Adjusted Δ** | **95% CI** | **P** |
| --- | --- | --- | --- |
| Primary multiple-imputation analysis | −4.40 | −7.30 to −1.50 | 0.003 |
| Complete-case analysis | −4.30 | −7.40 to −1.20 | 0.007 |
| Propensity overlap-weighted analysis | −4.20 | −7.10 to −1.30 | 0.005 |
| Conventional multivariable regression | −4.50 | −7.40 to −1.60 | 0.003 |
| Excluding patients who had surgery | −4.00 | −6.90 to −1.10 | 0.007 |
| Excluding major crossovers | −4.50 | −7.40 to −1.60 | 0.003 |
| Per-protocol (≥8 sessions) | −4.70 | −7.70 to −1.70 | 0.002 |
| As-treated analysis | −4.10 | −7.00 to −1.20 | 0.006 |
| Conservative worst-case imputation | −3.10 | −6.20 to 0.00 | 0.051 |
| Pattern-mixture (missing 2 points worse) | −3.70 | −6.70 to −0.70 | 0.016 |

**Recurrence sensitivity analyses (adjusted HR; 95% CI):** primary Cox 0.55 (0.34–0.89); overlap-weighted 0.53 (0.32–0.88); complete event-status cases 0.56 (0.34–0.92); excluding crossovers 0.51 (0.31–0.86); surgery as competing event 0.58 (0.36–0.94). **Missingness diagnostics:** overall missing 24-month ODI 34/208 (16.3%); combined 16/106 (15.1%); needling 18/102 (17.6%); adjusted OR for association of cohort with missingness 0.86 (0.41–1.78), P = 0.681; 50 imputed datasets, 20 iterations each.

**Table S12.** Exploratory subgroup analyses for 24-month ODI

| **Subgroup** | **Adjusted Δ** | **95% CI** | **Interaction P** |
| --- | --- | --- | --- |
| Age <45 years | −4.60 | −8.00 to −1.20 | 0.83 |
| Age ≥45 years | −4.10 | −8.30 to +0.10 |  |
| Male | −4.00 | −7.60 to −0.40 | 0.65 |
| Female | −4.80 | −8.80 to −0.80 |  |
| Symptom duration <6 months | −4.20 | −7.80 to −0.60 | 0.78 |
| Symptom duration ≥6 months | −4.70 | −8.60 to −0.80 |  |
| Single-level LDH | −4.80 | −8.00 to −1.60 | 0.54 |
| Multilevel LDH | −3.70 | −8.70 to +1.30 |  |
| Baseline ODI <30 | −3.50 | −6.90 to −0.10 | 0.21 |
| Baseline ODI ≥30 | −5.40 | −9.60 to −1.20 |  |
| Protrusion | −4.20 | −7.40 to −1.00 | 0.68 |
| Extrusion/sequestration | −4.90 | −9.80 to 0.00 |  |
| Baseline sagittal imbalance present | −5.10 | −8.90 to −1.30 | 0.30 |
| No predefined sagittal imbalance | −3.60 | −7.50 to +0.30 |  |

Associations were broadly consistent across subgroups; all interaction P > 0.05, so no subgroup should be claimed to benefit differentially. Subgroup analyses were exploratory.

**Additional materials added during revision**

The following items were added or corrected in response to peer review and editorial assessment: the literature search strategy; a completed STROBE checklist; the estimand and outcome-specific handling of crossover, surgery and missing data (Table S13); corrected absolute-benefit and number-needed-to-treat estimates (Table S14); an E-value sensitivity analysis (Table S15); tipping-point, landmark-recurrence and inverse-probability-weighted responder sensitivity analyses (addendum to Table S11); a summary of the reproducible statistical specification; and forest and propensity-score-overlap figures (Supplementary Figures S1 and S2). The full statistical analysis plan and annotated analysis code are provided as separate Supplementary files.

**Literature search strategy**

This was a targeted contextual search to identify background and comparative evidence, not a systematic review; no PRISMA flow or screening counts are therefore reported. Databases were searched from inception to the last-search date of 15 July 2026, with no language restriction. The reproducible core string was: (“lumbar disc herniation” OR “lumbar intervertebral disc” OR sciatica OR radiculopathy) AND (acupuncture OR “adjacent needling” OR needling OR “spinal manipulation” OR manipulation OR tuina OR “pelvic correction” OR “manual therapy”). Reference lists of retrieved reports and relevant guidelines were screened for additional studies.

| **Database** | **Coverage searched (last search 15 Jul 2026)** | **Field / language** |
| --- | --- | --- |
| PubMed / MEDLINE | Inception – 15 Jul 2026 | Title/abstract/keyword; no language limit |
| Embase | Inception – 15 Jul 2026 | Title/abstract/keyword; no language limit |
| Cochrane Library (CENTRAL) | Inception – 15 Jul 2026 | Title/abstract/keyword; no language limit |
| China National Knowledge Infrastructure (CNKI) | Inception – 15 Jul 2026 | Title/abstract/keyword; Chinese/English |
| Wanfang Data | Inception – 15 Jul 2026 | Title/abstract/keyword; Chinese/English |

**STROBE checklist for cohort studies**

The manuscript follows the STROBE recommendations for cohort studies; the location of each item is indicated below.

| **Item** | **STROBE recommendation** | **Location in manuscript** |
| --- | --- | --- |
| 1a | Study design in title/abstract | Title; Abstract (Methods) |
| 1b | Informative and balanced abstract | Abstract |
| 2 | Background and rationale | Introduction |
| 3 | Objectives / hypotheses | Introduction (final paragraph) |
| 4 | Study design | Methods, Study design and setting |
| 5 | Setting, locations, dates | Methods, Study design and setting |
| 6 | Eligibility, sources, selection | Methods, Participants and eligibility |
| 7 | Variables (outcomes, exposure, confounders) | Methods, Exposure, Outcomes, Statistical analysis |
| 8 | Data sources / measurement | Methods, Interventions, Radiographic, Laboratory |
| 9 | Bias | Methods, Statistical analysis; Discussion, Limitations |
| 10 | Study size | Methods, Sample size |
| 11 | Quantitative variable handling | Methods, Statistical analysis |
| 12 | Statistical methods; confounding; subgroups; missing data; sensitivity | Methods, Statistical analysis; Table S13 |
| 13 | Participants at each stage | Results, Participants; Figure 1; Table S1 |
| 14 | Descriptive data / missingness | Results; Table 1; Table S1 |
| 15 | Outcome data / events | Results; Tables 2–5 |
| 16 | Main results with adjusted estimates and CIs | Results; Tables 3–4 |
| 17 | Other analyses (subgroups, sensitivity, interactions) | Results; Tables S11, S12, S14, S15; Figures S1–S2 |
| 18 | Key results | Discussion (first paragraph) |
| 19 | Limitations | Discussion, Limitations |
| 20 | Interpretation; causation caution | Discussion; Conclusion |
| 21 | Generalisability | Discussion, Limitations |
| 22 | Funding | Declarations, Funding |

**Table S13. Estimand and outcome-specific handling of crossover, surgery and missing data**

| **Outcome** | **Time origin** | **Crossover** | **Surgery** | **Missing follow-up** |
| --- | --- | --- | --- | --- |
| ODI, VAS, JOA | Baseline | Analysed by initial cohort (as-treated in sensitivity analysis) | Post-surgery observations censored; pre-surgery retained | Mixed models with multiple imputation (50 datasets) |
| Symptomatic recurrence | Treatment completion (landmark, initial responders) | Retained by initial cohort | Competing event in sensitivity analysis; censored in primary | Censored at last contact |
| Additional conservative treatment | Baseline | Retained by initial cohort | Censored at surgery | Censored at last contact |
| Return to work | End of treatment | Retained by initial cohort | Censored at surgery | Questionnaire and record cross-check |

The primary estimand was the effect of the initial treatment strategy. “Intention-to-treat” terminology is reserved for randomised allocation and is not used. Informative censoring at surgery or crossover was examined in the competing-risk and as-treated sensitivity analyses (Table S11). ODI, Oswestry Disability Index; VAS, visual analogue scale; JOA, Japanese Orthopaedic Association.

**Table S14. Absolute benefit and number needed to treat at 24 months**

| **Outcome** | **Combined** | **Adjacent needling** | **Unadjusted difference** | **Descriptive NNT** | **Adjusted NNT (95% CI)** |
| --- | --- | --- | --- | --- | --- |
| ≥50% ODI improvement | 58.9% | 40.5% | 18.4 pp | 6 | 6 (3 to 41) |
| ≥30% ODI improvement | 73.3% | 57.1% | 16.2 pp | 7 | – |
| Recurrence-free at 24 months | 76.4% | 60.8% | 15.6 pp | 7 | 7 (5 to 30) |
| Return to usual work by 3 months | 84.1% | 70.9% | 13.2 pp | 8 | – |
| Sustained usual work at 24 months | 77.3% | 64.0% | 13.3 pp | 8 | – |

Descriptive NNT is the reciprocal of the unadjusted observed risk difference, rounded up; it is a descriptive count of how many patients would need to receive combined therapy for one additional observed outcome and is not an adjusted causal effect. Adjusted NNT is derived from the adjusted responder risk ratio and the adjusted 24-month cumulative recurrence and is shown only where a stable model-based estimate was available. pp, percentage points; ODI, Oswestry Disability Index; NNT, number needed to treat; CI, confidence interval.

**Table S15. E-value sensitivity analysis for unmeasured confounding**

| **Outcome** | **Adjusted estimate** | **E-value (point)** | **E-value (CI limit nearest null)** |
| --- | --- | --- | --- |
| 24-month ODI difference | −4.40 points | 2.39 | 1.57 |
| Symptomatic recurrence | HR 0.55 | 3.04 | 1.50 |
| Additional conservative treatment | HR 0.49 | 3.50 | 1.74 |
| ≥50% ODI responder | RR 1.41 | 2.17 | 1.31 |

The E-value is the minimum association (risk-ratio scale) an unmeasured confounder would need with both treatment and outcome, beyond measured covariates, to move the estimate (or its confidence limit) to the null. The point estimates showed moderate robustness to a single unmeasured confounder, whereas the confidence-limit E-values were smaller (about 1.3 to 1.7), indicating that more modest residual confounding could move some intervals to the null. E-values do not address selection, measurement, recall or model-specification bias. The continuous ODI estimate was converted to an approximate risk ratio before computing its E-value. HR, hazard ratio; RR, risk ratio; CI, confidence interval.

**Table S11 (addendum). Post hoc sensitivity analyses added during revision**

| **Analysis** | **Result** | **Interpretation** |
| --- | --- | --- |
| Delta-adjusted tipping point (primary 24-month ODI) | Significance lost at δ ≈ +2.6 ODI points per missing value in the combined cohort | Direction and magnitude preserved under most scenarios; not robust to strong MNAR |
| Landmark recurrence (initial responders; origin at treatment completion) | 24-month cumulative recurrence 22.7% (97 combined) vs 38.4% (86 needling); adjusted HR 0.56 (0.34–0.92) | Consistent with the all-enrolled recurrence analysis |
| ≥50% ODI responder under multiple imputation | Adjusted RR 1.39 (1.05–1.84) | Materially unchanged from complete-case |
| ≥50% ODI responder under IPO weighting | Adjusted RR 1.40 (1.05–1.87) | Materially unchanged from complete-case |

MNAR, missing not at random; IPO, inverse probability of observation; RR, risk ratio; HR, hazard ratio; ODI, Oswestry Disability Index. These analyses were added during revision and are post hoc.

**Reproducible statistical specification**

Analyses used R version 4.3.2 with packages lme4 (mixed models), survival and cmprsk (time-to-event and competing risks), PSweight (overlap weighting), mice (multiple imputation, 50 datasets), EValue (E-values) and sandwich (robust variance). The propensity score for combined therapy was estimated by logistic regression on baseline covariates: age, sex, body-mass index, manual occupation, symptom duration, recent analgesic use, previous disc-herniation episode, straight-leg-raise result, neurological deficit, herniation level, herniation morphology (protrusion versus extrusion/sequestration), multilevel disease, Pfirrmann grade, baseline ODI, VAS, JOA, lumbar lordosis, pelvic tilt and sacral slope, and the recorded principal reason for treatment choice. Treatment preference was encoded as a three-level categorical variable (clinician-identified lumbo-pelvic dysfunction, patient preference, other) rather than the fine-grained categories of Table S2, to avoid a near-deterministic model; overlap weights were used so that estimation was confined to the region of common support (Supplementary Figure S2). Overlap weights were combined with the mixed models by weighted estimation within each imputed dataset, and estimates were pooled by Rubin's rules with robust (sandwich) standard errors. Linear mixed models included random intercepts for participants with an unstructured covariance for the repeated ODI, VAS and JOA measures and fixed effects for cohort, time and their interaction. Proportional-hazards assumptions were checked with scaled Schoenfeld residuals, with no material violation. The principal practitioner was recorded but, being determined after treatment selection, was treated as a post-exposure variable and was not included as a baseline confounder. Full annotated code and the dated statistical analysis plan are provided as separate Supplementary files.

**Supplementary Figure S1. Exploratory subgroup analyses for the 24-month ODI difference**

Forest plot of the adjusted between-group difference in 24-month ODI (combined therapy minus adjacent needling) within subgroups defined by age (<45 vs ≥45 years), sex, symptom duration (<6 vs ≥6 months), herniation extent (single vs multilevel), herniation morphology (protrusion vs extrusion/sequestration), baseline ODI (<30 vs ≥30 points) and baseline sagittal imbalance (present vs absent). Squares are subgroup point estimates; the diamond is the overall estimate; horizontal lines are 95% confidence intervals. All interaction P values exceeded 0.05. These analyses were exploratory. Values correspond to Supplementary Table S12. ODI, Oswestry Disability Index; CI, confidence interval.

**Supplementary Figure S2. Propensity-score distribution and overlap**

Distribution of the estimated propensity score for combined therapy in each cohort, before and after overlap weighting, with the region of common support indicated. The distributions overlap across the full range with no evidence of positivity violation, and overlap weighting concentrates estimation in the region of common support. This supports the covariate-balance results, in which all weighted standardized mean differences were 0.05 or below.
