# Supplementary material for: Clinical outcomes associated with adding pelvic correction manipulation to adjacent needling for lumbar disc herniation: a prospective comparative cohort study
Source: Front Med (Lausanne). 2026 Aug 24;13:1936013. doi: 10.3389/fmed.2026.1936013 (PMC13547943; doi:10.3389/fmed.2026.1936013)
Supplement: Supplementary file 2 [file Data_Sheet_2.docx]

###############################################################################

```
## Analysis code
## Study: Adding pelvic correction manipulation to adjacent needling for
##        lumbar disc herniation - prospective comparative cohort (ID 1936013)
##
## This script documents the analysis pipeline referenced in the manuscript and
## the Statistical Analysis Plan. Variable names correspond to the analysis
## dataset (available under the data-sharing procedure in the manuscript).
## Original analysis: R 4.3.2. Post hoc additions (Section 8) were added during
## revision and are labelled as such.
```

###############################################################################

\## ---- 0. Packages -----------------------------------------------------------

library(lme4)	# linear mixed-effects models

library(survival)	# Cox proportional-hazards models

library(cmprsk)	# competing-risk (Fine-Gray) analysis

library(PSweight)	# propensity-score overlap weighting

library(mice)	# multiple imputation by chained equations

library(sandwich)	# robust (sandwich) covariance

library(lmtest)	# coeftest with robust SEs

library(EValue)	# E-values for observational estimates

set.seed(20201120) # reproducibility (plan finalisation date)

```
## ---- 1. Data ---------------------------------------------------------------
## dat: one row per participant, wide baseline + long outcome files joined.
## key columns:
##   cohort  : factor c("combined","needling")   (initial treatment strategy)
##   pref3   : factor 3-level treatment-preference encoding (avoids near-
##             deterministic propensity model)
##   odi0, odi_end, odi_24 ; vas*, joa* ; recurrence (0/1), t_recur ; ...
```

dat <- readRDS("analysis_dataset.rds")

\## ---- 2. Propensity score + overlap weights ---------------------------------

ps_formula <- cohort ~ age + sex + bmi + manual_job + duration + analgesic +

prior_ldh + slr + neuro_deficit + level + morphology + multilevel +

pfirrmann + odi0 + vas0 + joa0 + lordosis0 + pelvic_tilt0 + sacral_slope0 +

pref3

\## Overlap weights target the population of clinical equipoise.

ow <- PSweight(ps.formula = ps_formula, yname = "odi_change",

data = dat, weight = "overlap")

summary(ow)	# ATO estimate + balance

bal <- SumStat(ps.formula = ps_formula, data = dat, weight = "overlap")

plot(bal, type = "SMD")	# weighted standardized mean differences (<=0.05)

plot(bal, type = "density")	# propensity overlap -> Supplementary Figure S2

\## ---- 3. Missing data: multiple imputation (50 datasets) --------------------

imp <- mice(dat, m = 50, method = "pmm", seed = 20201120, printFlag = FALSE)

```
## ---- 4. Primary analysis: weighted linear mixed model ----------------------
## Fit within each imputed dataset with overlap weights, pool by Rubin's rules,
## robust SEs. Long-format repeated ODI (baseline, end, 24 months).
```

fit_one <- function(d){

dl <- to_long(d)	# helper: wide -> long repeated measures

m <- lmer(odi ~ cohort * time + covars + (1 | id),

data = dl, weights = ow_weight,

control = lmerControl(optimizer = "bobyqa"))

list(coef = fixef(m), vcov = sandwich::vcovCL(m, cluster = dl$id))

}

fits	<- lapply(complete(imp, "all"), fit_one)

pooled <- pool_manual(fits)	# Rubin's rules on coef/vcov

print(pooled)	# adjusted between-group ODI difference

\## ---- 5. Time-to-event: recurrence, additional treatment, surgery -----------

cox_recur <- coxph(Surv(t_recur, recurrence) ~ cohort + covars,

data = dat, weights = ow_weight, robust = TRUE)

cox.zph(cox_recur)	# proportional-hazards diagnostics

\## competing-risk sensitivity (surgery as competing event):

crr(ftime = dat$t_recur, fstatus = dat$status_cr, cov1 = model.matrix(~ cohort, dat)[,-1])

```
## ---- 6. Prespecified sensitivity analyses ----------------------------------
## complete-case, per-protocol, as-treated, worst-case pattern-mixture,
## conventional multivariable adjustment (code analogous to Section 4-5).
## ---- 7. E-values -----------------------------------------------------------
```

evalues.OLS(est = -4.40, se = 1.48, sd = 9.5)	# 24-month ODI difference

evalues.HR(0.55, lo = 0.34, hi = 0.89, rare = FALSE) # recurrence

evalues.RR(1.41, lo = 1.06, hi = 1.87)	# >=50% responder

```
## ---- 8. Post hoc additions (revision, July 2026) ---------------------------
## 8a. Delta-adjusted tipping point for the primary ODI outcome.
```

tipping <- function(delta){

di <- shift_imputed(imp, group = "combined", delta = delta) # worsen imputed ODI

est <- refit_primary(di)

est$p	# returns pooled P value

}

deltas <- seq(0, 4, by = 0.1)

tp <- sapply(deltas, tipping)

deltas[min(which(tp >= 0.05))]	# ~2.6 ODI points: significance lost

\## 8b. Landmark recurrence among documented initial responders.

resp <- subset(dat, initial_improved == 1)	# 97 combined, 86 needling

resp$t0 <- resp$t_treatment_completion	# time origin = completion

cox_lm <- coxph(Surv(t_recur_landmark, recurrence) ~ cohort + covars,

data = resp, weights = ow_weight, robust = TRUE)

summary(cox_lm)	# adjusted HR 0.56 (0.34-0.92)

```
## 8c. Number needed to treat.
## descriptive (unadjusted proportions, rounded up):
```

ceiling(1 / c(odi50 = .184, odi30 = .162, recfree = .156, rtw = .132, work = .133))

\## adjusted (model-based) responder and recurrence NNTs with CI:

nnt_from_rr <- function(rr, lo, hi, p0) ceiling(1 / (p0 * (c(rr, lo, hi) - 1)))

nnt_from_rr(1.41, 1.06, 1.87, p0 = 0.405)	# ~6 (3 to 41)

\## 8d. Responder outcome under MI and IPO weighting (vs complete-case).

glm_mi <- with(imp, glm(resp50 ~ cohort + covars, family = binomial,

weights = ow_weight))

pool(glm_mi)

```
## ---- 9. Figures ------------------------------------------------------------
## Figure 4 forest, Figure 6 panels, Supplementary Figures S1-S2 generated from
## the pooled estimates above (plotting code omitted for brevity).
```

###############################################################################
